# Development of Low Fluorinated, Sustainable, and Recyclable Electrolytes Based on γ‐Valerolactone for High‐Performance Sodium‐Ion Batteries

**DOI:** 10.1002/smll.202600061

**Published:** 2026-02-22

**Authors:** Yiyue Lu, Muhammad Nouman Aslam, Juan Luis Gómez Urbano, Shuting Zhang, Maider Zarrabeitia, Timo Werner, Peter Axmann, Christian Leibing, Andrea Balducci

**Affiliations:** ^1^ Institute of Technical and Environmental Chemistry Friedrich Schiller University Jena and Center for Energy and Environmental Chemistry (CEEC) Jena Jena Germany; ^2^ Helmholtz Institute Ulm (HIU) Ulm Germany; ^3^ Karlsruhe Institute of Technology (KIT) Karlsruhe Germany; ^4^ ZSW Center for Solar Energy and Hydrogen Research Baden‐Württemberg Ulm Germany

**Keywords:** sodium bis(fluorosulfonyl)imide, sodium difluoro(oxalato)borate, sodium‐ion battery, sustainable electrolyte, γ‐Valerolactone

## Abstract

This study introduces a dual salt novel electrolyte for sodium‐ion batteries (SIBs), consisting of sodium difluoro(oxalato)borate (NaDFOB) and sodium bis(fluorosulfonyl)imide (NaFSI) salts dissolved in the bio‐based γ‐valerolactone (GVL) solvent. Besides its renewable origin, the electrolyte exhibited strong inhibition of anodic dissolution and excellent electrochemical stability (up to 4.3 V vs. Na^+^/Na). It delivered outstanding cycling stability, with ∼87 % capacity retention after 100 cycles in P2‐Na_2/3_Al_1/9_Fe_1/9_Mn_2/3_Ni_1/9_O_2_ (P2‐AFMNO) cathode half cells and ∼80 % retention after 200 cycles in lab‐ scale full cells with hard carbon anodes when cycled within a wide voltage window of 1.5–4.3 V. Post mortem X‐ray photoelectron spectroscopy analysis helped gaining deeper understanding about the decomposition products formed on the interphases. A simple and sustainable water‐based process is employed to successfully recover the GVL solvent. The recovery method enabled recover 85 % of GVL solvent from the recycling process. The feasibility of recycling is further demonstrated by reusing the recovered GVL‐based electrolyte in full cells, which achieved performance comparable to that of the pristine GVL‐based electrolyte and exhibited excellent long‐term stability, retaining approximately 83 % of its capacity after 100 cycles.

## 1 Introduction

Sodium‐ion batteries (SIBs) are regarded as a promising alternative to lithium‐ion batteries (LIBs) owing to their low cost, abundant resources, and potentially lower toxicity [1, 2]. Significant progress has been achieved in recent years in improving the cycling stability and capacity retention of these devices, and SIBs are now commercially available [3, 4, 5, 6, 7].

Typically, SIBs employ hard carbon (HC) as an anodic material [8] while Prussian blue analogs [9, 10], polyanionic compounds [11, 12], or layered oxides [13, 14] are used as cathodic materials. Sodium hexafluorophosphate (NaPF_6_) in ethylene carbonate/propylene carbonate (EC/PC) or EC/dimethyl carbonate (DMC) remains the most common electrolyte system [15].

Although these devices already perform well, further efforts are needed to increase their overall sustainability. To achieve this goal, the development of innovative electrolytes with lower fluorine content and greater sustainability than the state‐of‐the‐art is of great importance. This is because the carbonate solvents (cyclic and linear) used to produce these electrolytes are obtained from non‐renewable fossil precursors, such as natural gas and crude oil [16, 17]. Additionally, the high fluorine content of the state‐of‐the‐art salt (NaPF_6_) dissolved in these carbonate solvents favors the formation of an effective solid‐electrolyte interphase (SEI), yet it also raises significant environmental and safety concerns [15, 18, 19].

Recently, we showed that the combined use of sodium bis(fluorosulfonyl)imide (NaFSI) and sodium difluoro(oxalato)borate (NaDFOB) in PC enabled the formation of stable SEI and cathode‐electrolyte interphase (CEI) under reduced fluorine conditions while maintaining excellent electrochemical performance [20, 21]. More specifically, the fluorine content is 18 wt. % for NaFSI and 23 wt. % for NaDFOB, much less when compared to conventional NaPF_6_ salts (68 wt. %).Furthermore, we demonstrated that γ‐valerolactone (GVL) is a promising solvent for different energy storage devices, including supercapacitors, lithium‐ion capacitors, and LIBs [22, 23]. GVL can be readily synthesized from biomass‐derived feedstocks such as cellulose, hemicellulose, or carbohydrates [22, 24, 25]. Moreover, it offers attractive features including biodegradability, low toxicity, a wide liquid temperature window, and a high flash point, making it a suitable replacement for conventional carbonate‐based solvents [22, 26]. To the best of our knowledge, the possible use of GVL in SIBs has been discussed only in a work by Mogensen et al. [27]. However, a detailed investigation of the use of GVL in SIBs has not been reported so far.

It is important to mention that in addition to the development of green electrolytes, the sustainability of battery technologies must be pursued through holistic life cycle maconcerns and recycling plays a pivotal role in this context, as it enables the recovery of critical metals (e.g., lithium, cobalt, and nickel), mitigates environmental and safety concerns, and ensures regulatory compliance. Although recycling of SIBs has recently drawn increasing interest, most studies have focused on electrode materials [28, 29, 30], leaving electrolyte recycling largely unexplored. In a recent study dedicated to GVL‐based electrolytes it has been reported a straightforward and effective approach for simultaneous recovery of both salt and solvent from an electrolyte of 1 m lithium bis(trifluoromethanesulfonyl)imide (LiTFSI) in GVL with 2 wt. % vinylene carbonate (VC) [31]. In that work, LiTFSI was subsequently evaluated in an aqueous electrolyte for electrochemical double‐layer capacitor (EDLC) applications [31]. Furthermore, while this work demonstrated the re‐utilization of the recovered salt, the reuse of the solvent remained unexplored. From this perspective, the use of GVL in SIBs could also be favorable in view of the development of recyclable electrolytes.

Herein, we report an investigation into the development of low‐fluorinated, sustainable, and recyclable electrolytes for SIB based on NaDFOB and NaFSI in GVL. The first part analyses in detail the thermal and transport properties of different GVL‐based electrolytes containing different ratios of NaDFOB and NaFSI, as well as their ability to suppress the anodic dissolution of aluminum current collectors. The use of all these electrolytes in combination with the P2‐Na_2/3_Al_1/9_Fe_1/9_Mn_2/3_Ni_1/9_O_2_ (P2‐AFMNO) cathode operating at 4.3 V vs. Na^+^/Na is also investigated in depth. In the second part, the most promising electrolyte formulation (0.8 m NaDFOB + 0.2 m NaFSI in GVL) is utilized for the realization of lab‐scale SIB full cells (HC || P2‐AFMNO). The impact of electrolyte degradation products on the compositional evolution of both SEI and CEI, as well as any associated morphological changes in the active material, is elucidated via post‐mortem analysis of cycled electrodes (1st and 200th cycles). The final part examines the recycling of this innovative electrolyte formulation. Using a water‐based method, GVL from cycled cells is recovered, purified, and reused in the HC || P2‐AFMNO laboratory scale SIB to verify the possibility of reusing this bio‐solvent and evaluate the impact on the cell performance.

## 2 Results and Discussion

### 2.1 Chemical‐Physical Characterization of the Electrolytes

**FIGURE 1:**
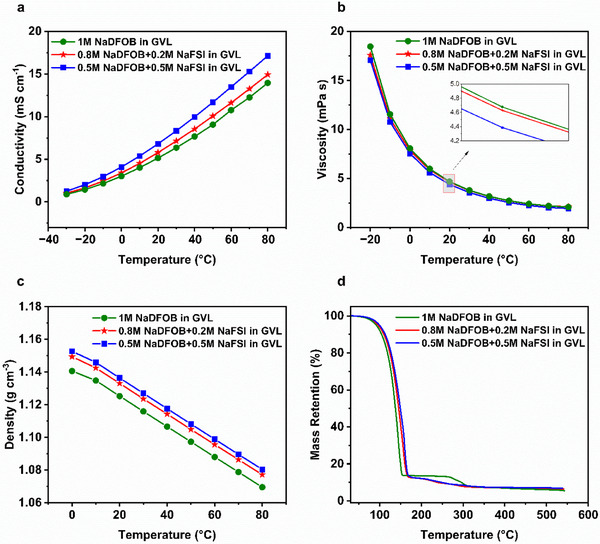
(a) Ionic conductivity between −30°C and +80°C; (b) viscosity between −20°C and +80°C; (c) density between 0°C and +80°C; (d) dynamic TGA curve in a temperature range from 30°C to 550°C of the investigated electrolytes, that is, 1 m NaDFOB in GVL (green), 0.8 m NaDFOB + 0.2 m NaFSI in GVL (red), and 0.5 m NaDFOB + 0.5 m NaFSI in GVL (blue).

Figure 1 compares the temperature dependence of ionic conductivity, viscosity, and density of the electrolytes 1 m NaDFOB in GVL, 0.8 m NaDFOB + 0.2 m NaFSI in GVL, and 0.5 m NaDFOB + 0.5 m NaFSI in GVL. As shown in Figure 1a, the electrolytes display comparable conductivity values across the entire temperature range. Nevertheless, higher conductivity values were measured upon increasing the NaFSI content in the formulations. More specifically, the conductivities at 20°C of 1 m NaDFOB in GVL, 0.8 m NaDFOB + 0.2 m NaFSI in GVL, 0.5 m NaDFOB + 0.5 m NaFSI in GVL, and 1 m NaFSI in GVL are 5.1, 5.8, 6.8, and 10.5 mS cm^−1^, respectively. The conductivity data for 1 m NaFSI in GVL are provided in Figure S1a. Corresponding values for PC‐based electrolytes employing the same salt systems and concentrations were 5.9 mS cm^−1^ for 1 m NaFSI in PC, 5.3 mS cm^−1^ for 0.8 m NaFSI + 0.2 m NaDFOB in PC, and 4.8 mS cm^−1^ for 0.5 m NaFSI + 0.5 m NaDFOB in PC at 20°C. These results demonstrate that the conductivity trend is mainly dependent on the salt choice [20]. However, it is interesting to note that GVL‐based electrolytes demonstrate distinctly higher ionic conductivity across the entire temperature range [20]. As shown in Figure 1b, the electrolytes also display very similar viscosity across the investigated temperature range. At 20°C, the viscosities of 1 m NaDFOB in GVL, 0.8 m NaDFOB + 0.2 m NaFSI in GVL, and 0.5 m NaDFOB + 0.5 m NaFSI in GVL are 4.67, 4.62, and 4.39 mPa s, respectively. These values are also slightly lower than those of electrolytes containing the same salts, but with PC as the solvent, for example, the viscosity of 0.5 m NaFSI + 0.5 m NaDFOB in PC is 6.62 mPa s [20]. Based on the literature‐reported solvation structure studies for these salts, it is suggested that in the single‐salt electrolyte (1 m NaDFOB in GVL), the strong Na^+^–DFOB^−^ pairing increases viscosity and limits free charge carriers. Incorporating NaFSI introduces the weakly coordinating, charge‐delocalized FSI^−^ anion, which replaces part of DFOB^−^ in the solvation shell, weakens Na^+^–DFOB^−^ interactions, and suppresses aggregation. This reorganization enhances Na^+^ mobility and reduces cluster size, leading to higher conductivity and lower viscosity in the dual‐salt electrolytes [20, 32, 33, 34]. It is also worth remarking that larger portions of NaFSI result in an increase in the electrolyte's density. Nevertheless, all formulations display densities within a narrow range between 1.12 and 1.13 g cm^−^^3^ at 20°C (Figure 1c).

Figure 1d compares the thermal stability of the three electrolytes studied. All electrolytes exhibit a significant mass loss at temperatures around 150°C, which can be mainly related to the evaporation of the GVL solvent. No significant mass loss is observed for the 1 m NaDFOB in GVL formulation between 150°C and 250°C. In contrast, dual‐salt electrolytes exhibit a gradual loss of mass from 160°C onward. This additional mass loss in the NaFSI‐containing electrolytes can be ascribed to differences in the salt‐solvent interaction and the evaporation of residual solvent. The observed temperature shift of approximately 10°C and the appearance of the second slope agree well with the isothermal measurements performed at 60°C (Figure S1c). It should be noted that the onset of NaFSI thermal decomposition occurs at around 200°C, as shown in Figure S1b. However, this initial decomposition does not lead to a pronounced mass loss and therefore overlaps with the gradual solvent‐related mass loss observed in the 160°C–210°C range. 1 m NaDFOB in GVL undergoes additional weight loss in the range of 270°C–300°C, which is associated with the thermal decomposition of NaDFOB (Figure S1b).

### 2.2 Electrochemical Characterization of the Electrolytes

**FIGURE 2:**
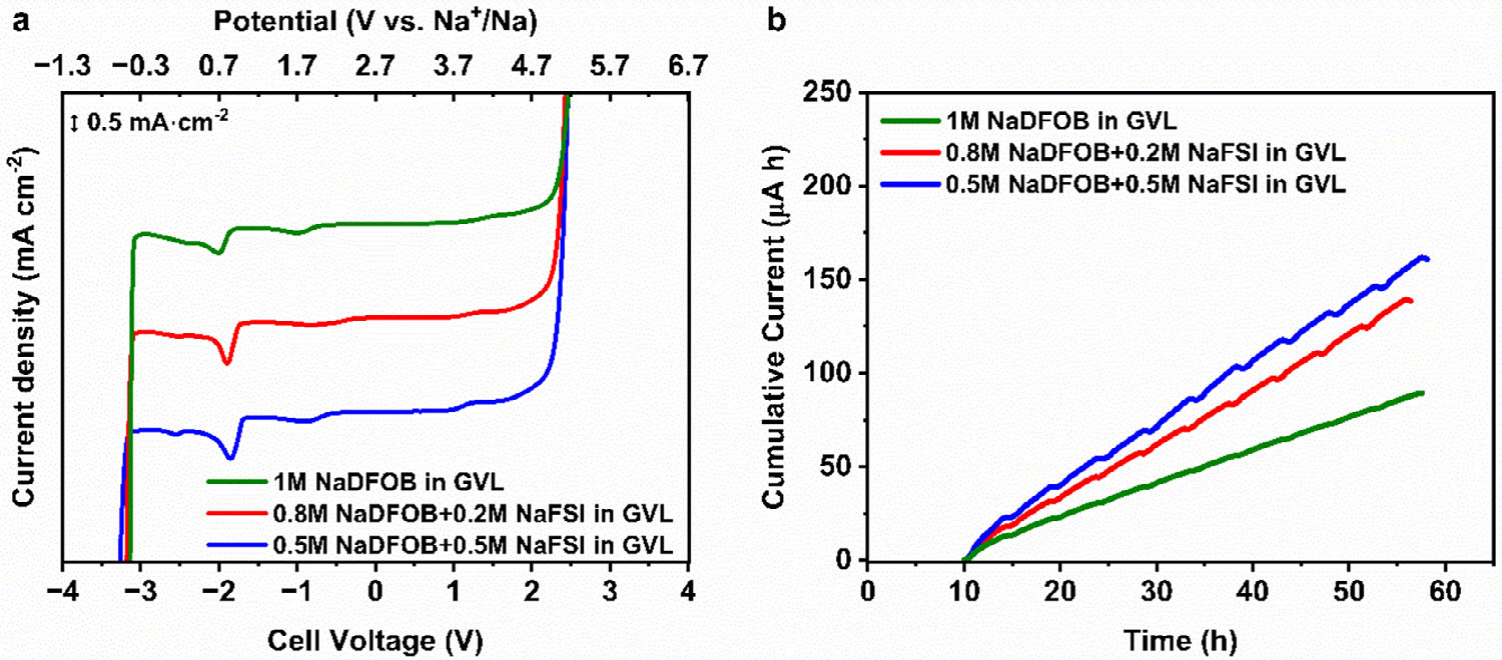
(a) Electrochemical stability window measured for the investigated electrolytes and (b) current evolution response from the anodic dissolution tests.

Figure 2a compares the electrochemical stability of the three electrolytes under investigation. As shown, all of them exhibit an oxidation stability of approximately up to 5 V vs. Na^+^/Na. The appearance of a peak at 0.7 V vs. Na^+^/Na, which is consistent with our previous results, is attributed to the onset of reductive instability of NaDFOB‐based electrolytes, which is associated with the reductive decomposition of the DFOB anion. Similar low‐potential irreversible reduction behavior has been widely reported for borate‐based salts, including Lithiumbis(oxalato)borat (LiBOB), and DFOB^−^ analogs, and is commonly correlated with anion decomposition and the formation of a passivation (SEI) layer [34, 35, 36]. It is worth noting that this low‐potential cathodic peak is absent in 1 m NaFSI in PC, as demonstrated in our previous work [20], indicating that the observed instability originates from NaDFOB rather than from the solvent or the NaFSI salt. As demonstrated by Bouchet's group, NaFSI‐ and Lithium bis(fluorosulfonyl)imide(LiFSI)‐based electrolytes exhibit wide electrochemical stability windows without a pronounced low‐potential cathodic decomposition peak [37]. In addition, a smaller cathodic peak observed at approximately 1.7 V vs. Na^+^/Na is also detected in NaDFOB‐containing electrolytes. This feature is generally attributed to trace oxalate species, such as residual oxalate esters or oxalate anions [34, 36]. However, the exact underlying reaction mechanism remains under debate, as these species constitute a mixture of oxalate‐derived components. For instance, Abraham's group proposed a ring‐opening reaction [38], while Sun's group showed that the observed species mainly arise from polymerization during reductive decomposition, supported by computational studies [39].

In view of the use of these electrolytes in combination with P2‐AFMNO cathodes, which operate at a potential at which anodic dissolution of aluminum current collectors can occur, it is important to elucidate their ability to suppress this harmful process. This is particularly relevant for NaFSI‐containing electrolytes due to the inability of this salt to prevent the corrosion of the aluminum current collector. Considering this, anodic dissolution tests were conducted to investigate electrolytes by potentiostatically scanning bare Al electrodes to potentials of 4.3 V vs. Na^+^/Na while registering the resulting evolution in current. As expected, the presence of NaFSI within the electrolyte leads to an increase in the cumulative current. The anodic dissolution measurements of 1 m NaFSI in GVL are described in Figure S2. However, as shown in Figure 2b, all electrolytes demonstrate a good ability to suppress this detrimental process, although none of them fully suppress it, as indicated by the F 1s region of the XPS spectra of Al discs after anodic dissolution tests, which demonstrates the formation of Al‐F species after cycling (Figure S3a,b). It is interesting to mention that the concentration of Al‐F species due to Al corrosion is reduced significantly when GVL solvent is used instead of PC (2.1 % vs. 7.6 %, respectively) [20]. Nevertheless, this increase is not dramatic, and no pitting was observed for Al current collectors after the test, indicating that the presence of NaDFOB is effectively protecting the Al surface.

### 2.3 Electrochemical Performance of P2‐AFMNO Cathodes

**FIGURE 3:**
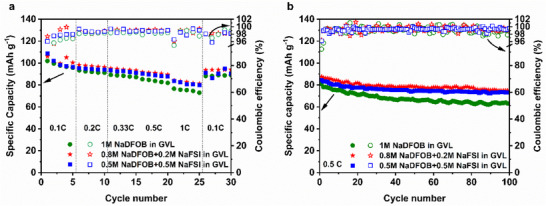
Comparison of (a) C‐rate capability test and (b) long‐term cycling at 0.5C of high voltage P2‐AFMNO cathode in the investigated electrolytes.

Figure 3a shows the rate performance of P2‐AFMNO electrodes cycled between 2.3 and 4.3 V vs. Na^+^/Na in the three GVL‐based electrolytes, while the voltage profiles at various current densities are provided in Figure S5. It should be noted that 1 m NaFSI in GVL was also investigated in P2‐AFMNO half cells. However, the cell fails to operate reversibly at 3.8 V vs. Na^+^/Na (see Figure S4). Therefore, the NaDFOB‐based electrolytes are demonstrated. At 0.1C, the electrode cycled in 1 m NaDFOB in GVL delivered a discharge capacity of 102 mAh g^−1^. The presence of NaFSI in the electrolyte enhanced the electrochemical performance: On the one hand, the P2‐AFMNO electrodes cycled in 0.8 m NaDFOB + 0.2 m NaFSI in GVL and 0.5 m NaDFOB + 0.5 m NaFSI in GVL display slightly higher discharge capacities of 106 and 108 mAh g^−1^, respectively 0.1C. On the other hand, the dual‐salt electrolytes also improved the rate capability in P2‐AFMNO half‐cells. At 1C, the P2‐AFMNO electrodes display discharge capacities of approximately 80 mAh g^−1^, which correspond to 80 % of the capacity at 0.1C. These results indicate that all electrolytes guarantee good capacity performance even at elevated rates. For more detailed half‐cell rate performance analysis, see Figure S5.

The long‐term cycling of GVL‐based electrolytes was evaluated in combination with P2‐AFMNO electrodes for 100 charge–discharge cycles at 0.5C (Figure 3b). Corresponding voltage profiles are provided in Figure S6. The 1 m NaDFOB in GVL formulation retained 77 % of its initial capacity after 100 cycles. In contrast, P2‐AFMNO electrodes cycled in electrolytes containing NaFSI demonstrated superior capacity retention, with values of 87 % and 85 % obtained for 0.8 m NaDFOB + 0.2 m NaFSI in GVL and 0.5 m NaDFOB + 0.5 m NaFSI in GVL, respectively. The observed capacity retentions indicate that although NaDFOB facilitates the formation of a stable CEI due to the formation of BOB^−^Mn(II) species (as observed in our previous work and others) [20, 40], which mitigates Mn dissolution, and suppresses dendrite growth [20, 21, 41], the use of a single salt cannot ensure long‐term electrochemical stability. For more detailed half‐cell long‐term cycling analysis, see Figure S6.

### 2.4 Electrochemical Performance of SIB Full Cell

**FIGURE 4:**
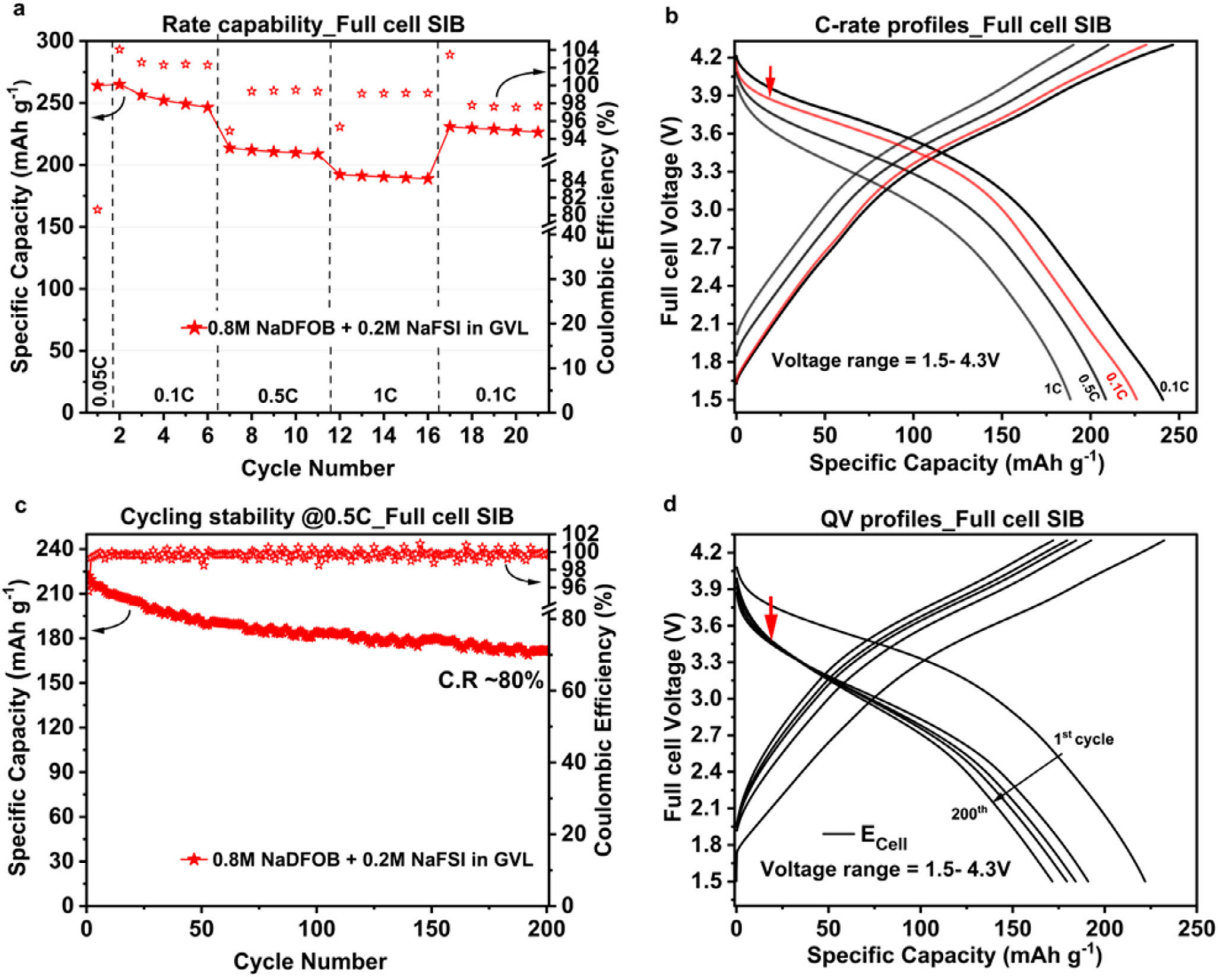
Investigations on electrochemical performance of HC || P2‐AFMNO SIB full cell (N:P = ∼1:1.5), (a) rate performance evaluation (first formation cycle at 0.05C followed by 5 cycles of charging‐discharging per each C‐rate), (b) C‐rate profiles representing cell voltage vs. specific capacity at different applied currents, (c) long cycling stability test conducted at 0.5C (after first formation cycle at 0.05C and 4 cycles at 0.1C), (d) Evolution of cell voltage vs. capacity profiles during the long cycle stability test at 0.5C over 200 cycles (1st, 50th,100th, 150th, and 200th), using 0.8 m NaDFOB + 0.2 m NaFSI in GVL electrolyte.; C.R = capacity retention (measured for 1st cycle vs. 200th cycle at 0.5C).

Based on the promising results obtained with the P2‐AFMNO cathode, the electrolyte 0.8 m NaDFOB + 0.2 m NaFSI in GVL was selected for the realization of lab‐scale full cell SIBs containing HC anode. The specific capacity values derived from the electrochemical half‐cell characterization of both positive (P) and negative (N) electrodes were used to calculate the capacity balancing of the SIB full cell. Detailed information on capacity balancing ratios for the full cells is provided in Figure S7. More specifically, two N:P capacity balancing ratios (∼1:1.5 and ∼1:1.55) were evaluated. It is important to mention here that, considering the individual electrode's capacity (in mAh) in these full cell evaluations, the HC electrode is a capacity‐limiting electrode while the cathode is an over‐capacity electrode. The capacity of the P2‐AFMNO cathode was oversized to effectively compensate for the lost Na^+^ during the initial few cycles of galvanostatic charge–discharge. This loss of Na^+^ stems from the lower initial Coulombic efficiency (ICE) observed in the HC anodes and is primarily attributed to the irreversible processes such as SEI formation on the HC surface, as well as irreversible binding of Na^+^ inside the HC host structure [42, 43, 44]. More in detail, the full cells assembled with an N:P capacity ratio of ∼1:1.55 exhibited reduced cycling stability and pronounced capacity decay relative to the cells with a ratio of ∼1:1.5, as evidenced by the results presented in Figure S8. Considering this, a detailed discussion related to the full cell with ∼1:1.5 ratio is presented in the following sections. The results of the investigations of the electrochemical performance of the lab‐scale SIB full cell are presented in Figure 4. Figure 4a presents the variation of the cell capacity at different C‐rates. As shown, the SIB full cell displays high capacity across all the applied C‐rates. At 1C, the full cell is delivering a capacity of ca. 190 mAh g^−1^, which increases to 230 mAh g^−1^ when the C‐rate is restored to 0.1C. The galvanostatic charge–discharge profiles recorded at different C‐rates for the lab‐scale SIBs are shown in Figure 4b. An overpotential induced by the application of higher currents at 1C is likely responsible for the small difference observed in the specific capacity at 0.1C (in black and red).

Regarding cell reversibility, the coulombic efficiency (CE) values for the lower C‐rate (0.1C) range between 97 % and 98 %, while for the higher C‐rates (0.5C and 1C), values above 99 % are calculated. It is important to notice that the C‐rate capability of the investigated full cell is fully comparable to that of a cell containing an electrolyte having the same salts but PC as solvent (see Figure S9), suggesting the sustainable GVL solvent could replace the PC carbonate solvent successfully [21]. The long‐term cycling stability of the full cell was investigated by carrying out 200 charge–discharge cycles at a constant rate of 0.5C. As shown in Figure 4c, at the end of the cycling process, the cell retained 80 % of its initial capacity (219 mAh g^−1^ at first cycle vs. 174 mAh g^−1^ at 200th cycle). It is worth noting that the cell maintained an average CE of > 99.6 % throughout the entire cycling test. Figure 4d shows the evolution of the galvanostatic charge–discharge profiles of the SIB full cell throughout the cycling process. A capacity loss of approximately 30 mAh g^−1^ was observed during the first 50 cycles. This gradual capacity decay is likely attributed to the increased overpotential, suggesting side reactions [45, 46] during the discharge process, indicated by the red arrow in Figure 4d. After initial 50 cycles, the full cell exhibits stable cycling with minimal capacity fade; specifically, the absolute capacity loss between the 50th and 200th cycles was calculated to be ∼16 mAh g^−1^ (representing a loss of only 8.5 % relative to the 50th cycle), suggesting that increment in overpotential is negligible with indication of stable and robust interphases formed on the electrode surfaces, contributing to the extended cycle life of the SIB full cell. These results indicate that the use of 0.8 m NaDFOB + 0.2 m NaFSI in GVL enables the realization of high‐performance SIBs. The cell exhibits capacity, capacity retention, and cycle stability that are perfectly comparable to those of SIBs containing PC‐based electrolytes [21]. This clearly demonstrates that the introduction of a low‐fluorinated GVL‐based electrolyte is possible and allows for an improvement in the overall sustainability of the system without negatively affecting cell performance. Further improvements could be achieved by optimizing the electrode mass loading and capacity balance (N:P ratio). Such optimization, however, is beyond the scope of this study.

### 2.5 Surface Characterization of Full Cell Electrodes

The excellent capacity retention of the SIB full cell based on HC ǁ 0.8 m NaDFOB + 0.2 m NaFSI in GVL ǁ P2‐AFMNO could be related to the formed interphases, that is, SEI and CEI, since previous works, including ours, indicated that the presence of DFOB^−^ anion could stabilize the interphase [20, 40]. Therefore, XPS was performed to elucidate the electrolyte decomposition mechanism and identify interphase chemical species. For that, the pristine, soaked, and cycled HC and P2‐AFMNO electrodes were analyzed by XPS at discharged states (i.e., after the 1st and 200th cycles). The chemical composition of the formed SEI and CEI on HC and P2‐AFMNO electrodes, respectively, was investigated by collecting the C 1s, F 1s, and B 1s photoelectron core‐level spectra.

**FIGURE 5:**
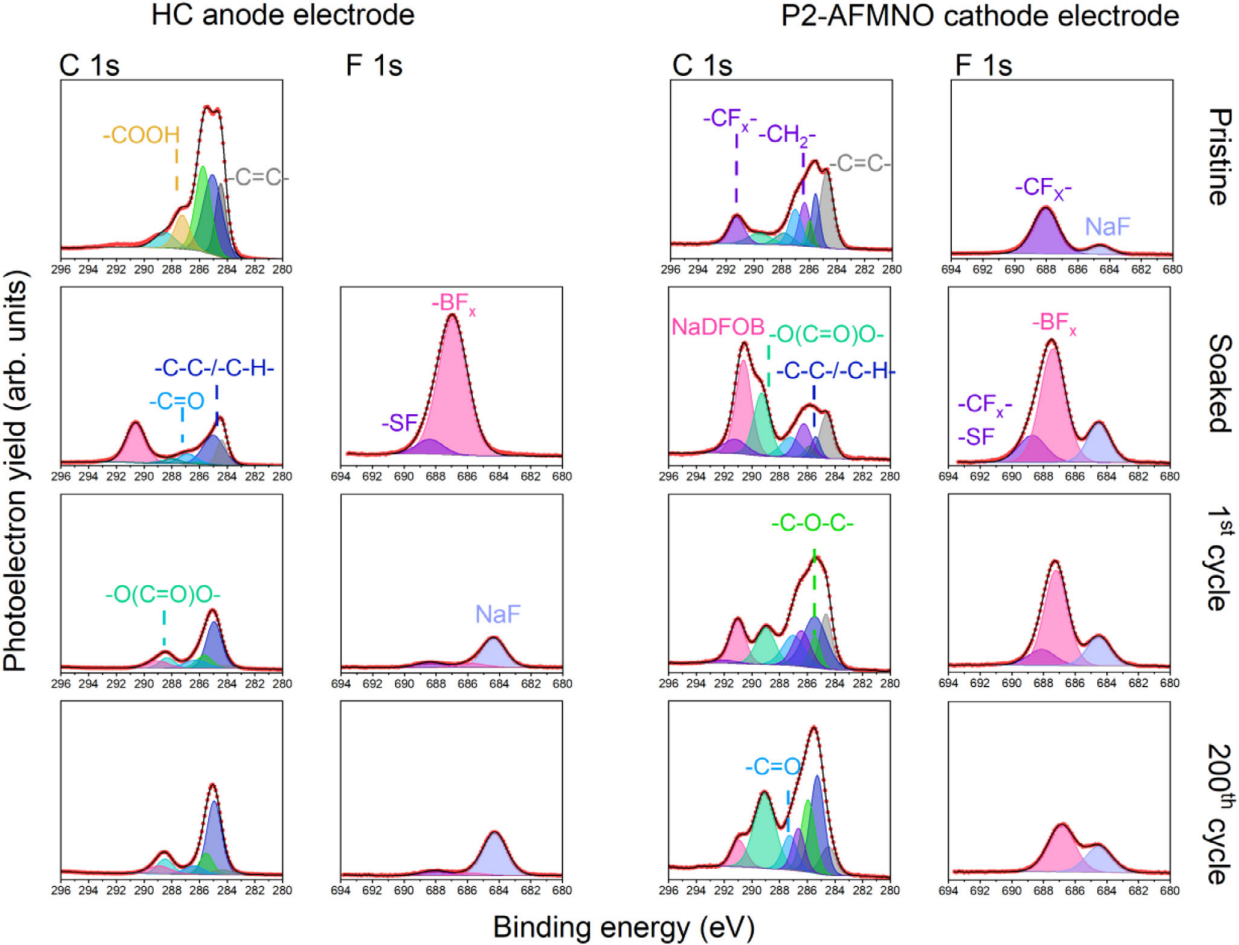
C1s and F 1s XPS core level spectra for HC (left) and P2‐AFMNO (right) electrodes before cycling (pristine and soaked) and after cycling at discharge states (1st and 200th cycle).

The C 1s spectra (Figure 5) of cycled HC electrodes clearly show the presence of electrolyte species and their decomposition products, as well as the decrease in concentration of the ─C═C─ bond at 284.4 eV, corresponding to the pseudo graphitic domains of HC, indicating the formation of the SEI. This is clear when the intensity of the ─C═C─ peak is compared with that in the pristine state. The formed SEI is mainly composed of hydrocarbons (─C─C─/─C─H─ at 284.8–285.0 eV), carbonyl (─C═O at ≈287.0 eV), and ester (─COOR) at ≈288.5 eV [47, 48], arising from the GVL and its reduction species, and ─O─(C═O)─(C═O)─O─ bond from the DFOB^−^ anion (at ≈289.0 eV). This indicates that the SEI has formed from the reduction of both GVL and NaDFOB. Regarding the HC electrode soaked in the electrolyte under study, the lower concentration of the ─C═C─ bond, corresponding to the electrode, might be due to the high amount of NaDFOB salt deposited on top of the electrode, as also suggested by the F 1s region (see the peak at ≈687 eV, corresponding to the B─F bond from NaDFOB). Interestingly, compared with the SEI formed in a similar electrolyte using a PC solvent rather than GVL [21], the formation of inorganic carbonates, such as Na_2_CO_3_ at >290 eV [47], is not observed, which is beneficial, as carbonates have been reported to dissolve in carbonate‐based electrolytes [49], potentially destabilizing the SEI upon cycling. This represents an advantage of the GVL‐based electrolyte. In fact, the SEI is mainly formed by hydrocarbons, and esters from GVL, as well as some ─O─(C═O)─(C═O)─O─ traces from DFOB^−^.

On the other hand, the species formed from the reduction of both salts, that is, NaDFOB and NaFSI, contribute to the formation of the SEI. These are clearly displayed in the F 1s and B 1s regions (see Figure S10). Both salts are decomposed, forming NaF (684.5 eV) and Na─B─O (≈192.5 eV) species. After soaking the HC electrode in the electrolyte, a large contribution from the NaDFOB salt is observed; however, once the HC electrode is cycled, the salts decompose, forming a homogeneous concentration of NaF, which completely covers the Na─B─O species after 200 cycles, due to the still growing SEI as indicated by the SEM images below. Nevertheless, these results indicate that the formed SEI is rather stable in terms of chemical composition upon cycling, comprising only some hydrocarbon (see C 1s: 23.5 % vs. 33.3 % in the 1st and 200th cycles, respectively).

The decomposition of the electrolyte in the P2‐AFMNO cathode electrode has also been investigated by analyzing the C 1s, F 1s, and B 1s photoelectron regions. In the case of the CEI, the chemistry is rather similar to that of the SEI formed on the HC anode electrode. The CEI is also mainly formed by species with an O─(C═O)─(C═O)─O─ bond, as well as carbonyl (─C═O), ethers (─COC─), and esters (─COOR), from the GVL solvent. The main differences are the lower concentration of hydrocarbons (─C─C─/─C─H─) and higher concentration of carbonyl and ether‐based species, which is in line with the expectation to observe more oxidized and fewer reduced species. In addition, it should be considered that the formed CEI is thinner and or more heterogeneous than the SEI, since the ─C═C─ (at 284.4 eV), and ─CH_2_─/─CF_X_─ (286.4 and 291.9 eV) peaks [48, 50] corresponding to the conductive additive (C65) and binder (PVDF) of the P2‐AFMNO electrode, are observable, even after 200 cycles.

Despite some differences in the C 1s region, the F 1s and B 1s photoelectrons show more drastic variations. The results clearly demonstrate that the SEI is mainly composed of NaF from the reduction of NaDFOB and NaFSI, while in the CEI, the NaDFOB is not easily decomposed, still showing a high concentration of ─BF_X_ bonds and ─SF bond from NaFSI, as well as NaF. The combination of similar concentrations of NaF in the CEI and SEI, the presence of ─BF_x_ species, and the detection of pre‐existing NaF in the P2‐AFNMO soaked electrode indicate that the NaDFOB is preferentially reduced at the surface of the HC anode electrode. This is also consistent with our previous work using PC solvent [21], as well as suggesting that the NaF is mainly formed from the PVDF dehydrofluorination [49, 51].

**FIGURE 6:**
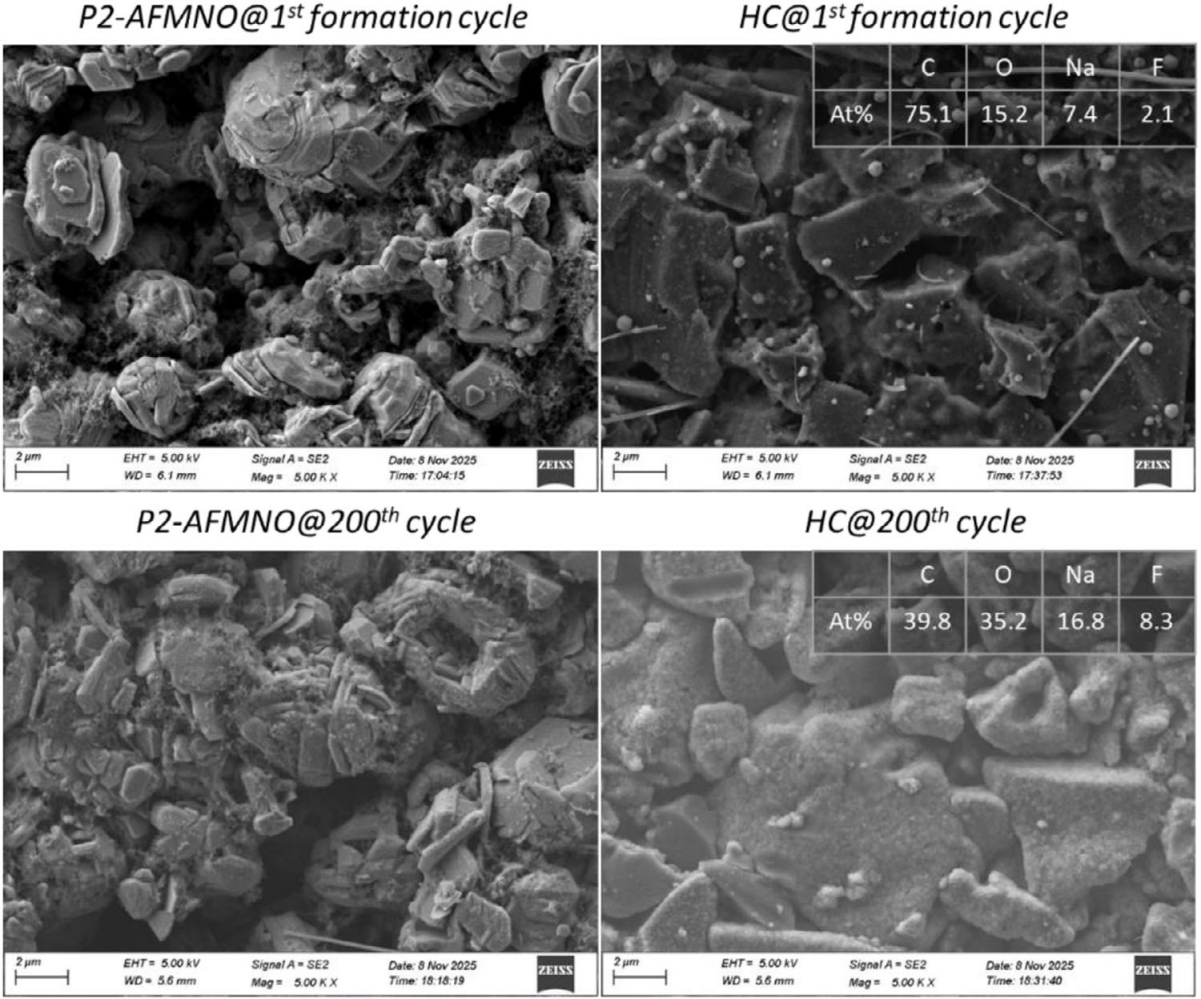
SEM images of the cycled electrodes in the full cell HC || P2‐AFMNO using the 0.8 m NaDFOB + 0.2 m NaFSI in GVL electrolyte. Estimated at % (via EDX) on the HC anode surface after the 1st and 200th cycles are shown in the upper‐right corner of the HC SEM images.

Regarding the morphological changes and species formed upon cycling of both electrodes, HC and P2‐AFMNO, scanning electron microscopy (SEM) and energy‐dispersive X‐ray spectroscopy (EDX) analyses have been conducted. As shown by the SEM (Figure 6) and EDX (Figure S11) images, the P2‐AFMNO cathode morphology remains stable upon cycling, with no significant changes after 200 cycles and thin surface layer formation, in agreement with the XPS results, which also indicated a thin CEI. The EDX mapping images (Figure S11) display a uniform distribution of elements with no significant changes upon cycling, indicating excellent structural and chemical stability of the P2‐AFMNO cathode.

**FIGURE 7:**
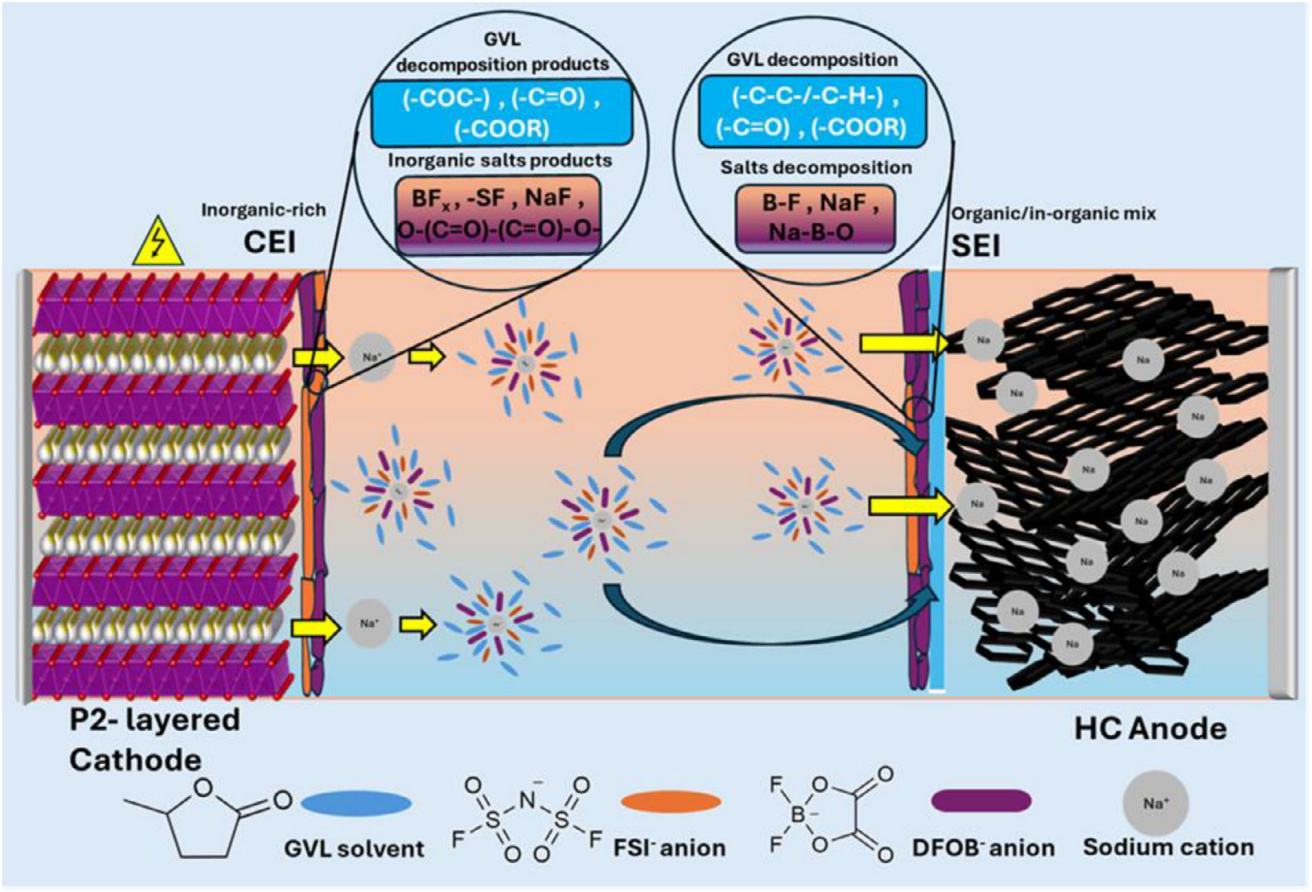
A schematic to understand dual‐salt electrolyte design and its correlation with the interphases (SEI/CEI) formed on the electrode surfaces.

In contrast, although the HC electrodes do not exhibit considerable morphological changes during cycling, SEM images clearly show SEI formation. Indeed, the EDX results reveal increased surface concentrations of O, F, and Na, while C content decreases, indicating progressive SEI growth, and in line with the XPS results. The illustration in Figure 7 correlates our dual‐salt electrolyte design with the chemical compositions of SEI/CEI layers determined by XPS, providing a clearer understanding of the interphase formation in this system.

### 2.6 Recycling and Reuse of GVL‐Based Electrolyte

As mentioned in the introduction, the recycling and reuse of electrolytes for LIBs employing GVL as a solvent have been demonstrated to be feasible [31]. Building upon this concept, we explore the possibility of recycling the GVL‐based electrolytes utilized for the fabrication of the lab‐scale SIB full cells discussed above. It is important to note that this study focuses exclusively on the recovery of the GVL solvent. To validate the feasibility of this approach, the recovery process was applied to both fresh and electrochemically aged GVL‐based electrolytes, and the “recycled GVL” was subsequently reused in its second life as a solvent for SIB full cells.

The recycling of the GVL from the electrolyte was performed following the procedure reported by Teoh et al. [31]. More in detail, a liquid‐liquid extraction was carried out to isolate the conductive salts in the aqueous phase, while the GVL solvent can be recovered from the organic phase together with the organic extractants employed (i.e., ethyl acetate and cyclohexane). After separation, the organic phase was subjected to vacuum distillation to obtain the volatile fraction (EtOAc, c‐Hex) and the distillation tail (GVL). The recovered distillation tail was subsequently dried overnight to ensure a proper removal of residual solvents using a Schlenk line under vacuum. The volatile fraction and the distillation tail were analyzed by nuclear magnetic resonance (NMR) spectroscopy (Figure S12). The volatile fraction mainly consisted of EtOAc and c‐Hex, retaining the initial 1:1 volume ratio, which was calculated based on the NMR data and compared with the corresponding theoretical value. A brief explanation has been added to the SI for clarification. (Figure S12a,b). In contrast, the ^13^C and ^1^H NMR spectra of the distillation residue (Figure S12c,d) indicated that it was predominantly composed of GVL, with almost no detectable EtOAc or c‐Hex. This suggests that the recovered GVL had a purity close to 100 %, which was further confirmed by TGA, see Figure S13. The recovery method recovers around 85 % of GVL solvents from the recycling process. These results demonstrate that this method can also be applied to recover sodium‐based electrolytes using GVL as the solvent. After removal of excess water with molecular sieves, a new electrolyte was prepared with fresh salts and the recycled GVL. The ^11^B and ^19^F NMR spectra of the aqueous phase (containing NaDFOB and NaFSI salts) are reported in Figure S14. Despite the absence of significant peaks related to decomposition products, salt recovery was not pursued in this study due to the susceptibility of these salts to hydrolysis, which merits deeper, independent studies.

**FIGURE 8:**
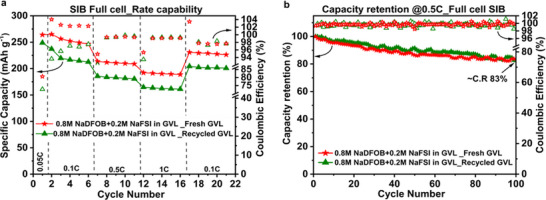
Investigations on electrochemical performance of HC || P2‐AFMNO full cell (N:P = ∼1:1.5), using 0.8 m NaDFOB + 0.2 m NaFSI in GVL electrolytes with “fresh GVL” (in red) and “recycled‐GVL” (in olive green). (a) C‐rate capability evaluation (first formation cycle at 0.05C followed by 5 cycles of charging‐discharging per each C‐rate), (b) capacity retention (%) after 100 cycles of galvanostatic charging‐discharging at 0.5C C‐rate; C.R = capacity retention (measured for 1st cycle vs. 100th cycle at 0.5C).

The “recycled GVL” solvent was recovered from electrochemically aged lab‐scale SIB cells containing 0.8 m NaDFOB + 0.2 m NaFSI in GVL electrolyte (details from the recovery of spent electrolytes from the lab‐scale cells are reported in the experimental section). To validate its reuse, the recycled GVL was used as a solvent to prepare the 0.8 m NaDFOB + 0.2 m NaFSI in GVL electrolyte. This new electrolyte, based on “recycled GVL,” was then directly employed in our lab‐scale SIB full cell. A comparison of the electrochemical performance of SIB full cells containing the “fresh” and “recycled” GVL solvents is presented in Figure 8. For clarity, the “fresh GVL” refers to the electrolyte prepared with pristine GVL solvent, whereas the “recycled GVL” denotes the electrolyte formulated using GVL solvent recovered from the electrochemically aged cells. The C‐rate capability performance is illustrated in Figure 8a, while the corresponding galvanostatic charge–discharge profiles for the “recycled GVL” based electrolyte are presented in Figure S15. The SIB full cell employing the recycled GVL‐based electrolyte displayed stable rate performance with consistent capacity across all the applied C‐rates. Despite slightly lower capacity values than those observed with the fresh electrolyte, the full cell maintained stable performance even at a 1C rate, delivering a capacity of approximately 160 mAh g^−1^. The SIB full cell exhibited a stable and reversible capacity of greater than 200 mAh g^−1^ when restoring the rate to 0.1C. The recorded CE values for the lower C‐rate (0.1C) range between 97 % and 98 % and for the higher C‐rates (0.5C and 1C), the CE values exceeded 99 %, very similar values to those obtained with “fresh GVL” electrolyte. At the beginning of the rate test, the CE of the “recycled GVL” is slightly lower, which could be related to the presence of small impurities in the recovered GVL. This led to a small decrease in capacity due to the irreversible loss of Na^+^ ions. Nevertheless, future studies will be directed to further purify GVL solvents, and this can also be potentially alleviated by mixing recycled GVL with fresh GVL to ensure competitive performance and enough recycling content in the cell.

The long‐term stability performance of the “recycled GVL” electrolyte in the SIB full cell was evaluated over 100 galvanostatic charge–discharge cycles at 0.5C. This lab‐scale SIB full cell exhibited ∼83 % retention of its initial capacity (173 mAh g^−1^ at first cycle vs. 143 mAh g^−1^ 100th cycle), which is equivalent to that achieved with the “fresh GVL” electrolyte (see Figure 8b). These findings confirm the comparable electrochemical performance achieved with the “recycled GVL” based electrolyte, thereby validating the proof‐of‐concept for GVL solvent recycling, and its suitable secondary use in SIBs.

## 3 Conclusion

This study reports the development of a novel, sustainable, and recyclable GVL‐based electrolyte formulated with low‐fluorinated salts (NaDFOB and NaFSI). Besides its greener profile, this electrolyte offers superior safety and thermal stability, which was confirmed by TGA. Its ability to suppress the anodic dissolution is further highlighted by the low concentration of Al‐F species measured by XPS, ensuring good compatibility with high‐voltage P2‐AFMNO layered oxide cathodes. This sustainable electrolyte exhibited very high stability for the P2‐AFMNO cathode in half‐cells, retaining approximately 87 % of its capacity after 100 cycles when cycled up to 4.3 V vs. Na^+^/Na. Furthermore, our laboratory‐scale SIB demonstrator full cell (HC || P2‐AFMNO) incorporating this novel electrolyte delivered superior electrochemical performance across a 1.5–4.3 V operating window, demonstrating ∼80 % capacity retention after 200 cycles. The surface chemistry, compositional, and morphological evolution of interphases (SEI and CEI) and electrode particle morphology were assessed by post‐mortem XPS/EDX and SEM analysis, respectively, before and after the stability testing of the full cell for 200 cycles. XPS investigations revealed the formation of homogeneous SEI and thin CEI, while SEM analysis confirmed minimal morphological changes of electrode particles after 200 cycles, mainly observing the formation of the SEI on top of the HC electrode. To further validate the proof‐of‐concept for GVL‐based electrolytes recycling in SIBs, the successful recycling and subsequent reutilization of recycled GVL‐based electrolytes were demonstrated in lab‐scale full cells. Consequently, these findings not only indicated that it is possible to successfully introduce GVL‐based electrolytes but also made a vital contribution to circular economy goals through the recycling and reuse of this electrolyte in SIBs.

## 4 Experimental Section

### 4.1 Electrodes Preparation

#### 4.1.1 Anode Electrode Preparation

HC electrode sheets were prepared by dispersing a commercial HC powder (Kuranode Hard Carbon Type 2, average particle size D_50_ = 5 µm, specific surface area = 6 m^2^ g^−1^, interlayer spacing d_002_ = 0.38 nm), sodium carboxymethylcellulose (Na‐CMC, SunroseMAC500, Nippon Carbon Co., Ltd.), and styrene–butadiene rubber (SBR, BM‐451B, Zeon) binders in deionized water. The components were mixed at a mass ratio of 94.6:1.8:3.6 (HC:Na‐CMC:SBR), yielding a homogeneous slurry. The slurry was subsequently coated onto aluminum current collector foils (Korff AG, thickness 15 µm) using the doctor‐blade method.

The HC electrodes intended for full cell assembly were prepared by using a wet‐film thickness of 180 and 200 µm, yielding an active material areal mass loading of approximately 6.30 and 7.15 mg cm^−^^2^, respectively. The dried electrodes were subsequently calendered with a lab‐calender (Sumeet GmbH) at 40°C under a line pressure of 3 N mm^−1^ and a roll speed of 1 m min^−1^ to optimize the electrode's surface uniformity.

#### 4.1.2 Cathode Synthesis and Electrode Preparation

P2‐Na_2_/_3_Al_1_/_9_Fe_1_/_9_Mn_2_/_3_Ni_1_/_9_O_2_ (referred to as P2‐AFMNO) was synthesized in our laboratory following a three‐step procedure: (i) precipitation of a spherical double‐layer hydroxide precursor with an Al:Fe:Mn:Ni molar ratio of 1:1:6:1; (ii) dry mixing of this precursor with Na_2_CO_3_ to achieve a Na:M ratio of 2:3 (M═Al, Fe, Mn, Ni); and (iii) calcination of the mixture at 950°C for 10 h in synthetic air (20 vol. % O_2_ in Ar). A detailed description of the synthesis route and characterization of the cathode active material is provided in Figure S16.

Following calcination, the resulting cathode material was immediately transferred to a Büchi glass oven and kept at 200°C under dynamic vacuum (∼2 × 10^−^^2^ Pa) overnight. All subsequent powder handling and electrode preparation were carried out in the same glovebox to avoid exposure to ambient air.

Cathode composite electrodes were fabricated by dispersing the active material, SuperP‐Li conductive carbon (Imerys Graphite and Carbon), and polyvinylidene difluoride (PVDF, Solvay Solef P5130) binder in a mass ratio of 93:3:4 in an appropriate amount of anhydrous N‐methyl‐2‐pyrrolidone (Sigma–Aldrich). The resulting slurry was cast onto aluminum current collector foils using the doctor blade technique. The dried electrode sheets were roll‐pressed using a laboratory calender (Sumeet) at 1 m/min with a line pressure of 10 N/mm at 100°C. Electrodes with a diameter of 12 mm were then punched from the sheets and further dried overnight at 80°C under dynamic vacuum (1 × 10^−1^ Pa) in a Büchi glass oven. The typical electrodes had an active material mass loading of ∼23.2 mg cm^−^^2^. SEM images of the electrode surface and cross‐sections are provided in Figures S17–S19.

### 4.2 Electrolyte Preparation

NaFSI (BLDpharm, 99.97 %) and NaDFOB (Sigma–Aldrich, ≥99 %) salts were used without further purification. γ‐Valerolactone (GVL, Sigma–Aldrich, anhydrous, ≥99 %) was pre‐dried over 3 Å molecular sieves for three days prior to use. Electrolytes were prepared inside an argon glovebox (MBraun, H_2_O< 5 ppm, O_2_< 5 ppm) by dissolving the required salt(s) in GVL under magnetic stirring.

### 4.3 Chemical‐Physical Characterization

#### 4.3.1 Conductivity

The ionic conductivity of the electrolytes was determined in a temperature range of −30°C–80°C by measuring the alternating current resistance (Modulab XM ECS potentiostat) of a cell with two parallel platinum electrodes and a known cell constant as described in the literature [52].

#### 4.3.2 Density

Electrolyte density was recorded between 0°C and 80°C using a DMA 4100 M density meter (Anton Paar) with 0.5 mL sample volume.

#### 4.3.3 Viscosity

Viscosity was measured using an MCR 102 modular rheometer (Anton Paar) from −20°C to 80°C at a shear rate of 1000 s^−1^, with 0.35 mL electrolyte samples. The measurements were carried out according to the procedure described in the literature [52].

#### 4.3.4 Thermal Stability

TGA was performed on a PerkinElmer STA 6000. The instrument was purged with nitrogen for 1 h before use. Measurements were conducted under nitrogen flow (30 mL min^−1^, 2.5 bar) from 30°C to 550°C at 10°C min^−1^. Additional isothermal tests were carried out at 60°C for 24 h.

### 4.4 Electrochemical Characterization

#### 4.4.1 Electrochemical Stability Window

Three‐electrode Swagelok‐type cells were assembled in an argon‐filled glovebox using a platinum disk as the working electrode, a self‐standing activated carbon disk as the counter electrode, and the reference electrode. Each cell contained 150 µL of electrolyte and a Whatman GF/D glass fiber separator. Linear sweep voltammetry was performed at 1 mV s^−1^ (±0.1 mA cm^−^^2^ current threshold) on a BioLogic VMP‐3 potentiostat.

#### 4.4.2 Anodic Dissolution

Three‐electrode Swagelok cells were used with aluminum foil as the working electrode, sodium metal as counter and reference electrodes, and 300 µL of electrolyte with GF/D separators. The potential was cycled between 2.3 and 4.3 V (vs. Na^+^/Na) at 0.5 mV s^−1^ for 10 cycles, with a 3 h hold at 4.3 V in each cycle.

#### 4.4.3 Galvanostatic Cycling

For the half‐cell and full cell electrochemical measurements, a 3‐electrode cell (Swagelok cell) setup was used. Half‐cells with P2‐AFMNO cathodes were assembled against sodium metal as counter and reference electrodes. Charge–discharge tests were conducted between 2.3 and 4.3 V (vs. Na^+^/Na) at rates of 0.1C, 0.2C, 0.33C, 0.5C, and 1C (1C = 75 mAh g^−1^), with five cycles per current rate. Long‐term stability was tested at 0.5C within the same voltage range.

For the lab‐scale SIB full cells, P2‐AFMNO cathode and HC anode were used as the positive and negative electrodes, respectively, with metallic Na serving as the reference electrode. The full cells utilized thicker electrodes, incorporating cathode active mass loadings of 19.47 and 21.36 mg cm^−2^ and corresponding HC anode active mass loadings of 6.30 and 7.15 mg cm^−2^. Two capacity balancing ratios N/P = ∼1:1.5 and N/P = ∼1:1.55, were investigated. The current density and specific capacity values reported for the full cell are based on the active material mass of the HC anode. The N/P ratio is determined from the reversible areal capacities (mAh cm^−2^) of the individual electrodes at a similar current density. The following equation is used to calculate the N/P balancing ratio [21, 53].

$$N/P\;\mathrm{ratio}=\frac{\mathrm{Anode}\;\mathrm{reversible}\;\mathrm{areal}\;\mathrm{capacity}\;}{\mathrm{Cathode}\;\mathrm{reversible}\;\mathrm{areal}\;\mathrm{capacity}}$$

where areal capacity units are mAh cm^−2^. Electrochemical testing of the full cells was performed between different cathode and anode potentials, 1.5 to 4.3 V. All measurements were performed at room temperature with 12 mm electrodes, 150 µL of electrolyte, and one (Whatman GF/D) glass fiber separator.

### 4.5 Surface Characterization

The morphology and the interphase chemistry of both cycled P2‐AFMNO cathode and HC anode electrodes after the 1st and 200th cycles were examined using high‐resolution scanning electron microscopy (FE‐SEM, ZEISS) and X‐ray photoelectron spectroscopy (XPS), respectively. The SEM images were collected with an acceleration voltage of 3 kV, and EDX was performed. The chemistry of the passivation layers and formed interphases was also investigated in pristine and soaked electrodes for comparison. The interphases were studied through the acquisition of high‐resolution C 1s, F 1s, and B 1s photoelectron region, using a monochromatic Al Kα source (hν = 1487 eV9 at 200 W, and 12 kV, with an energy pass of 0.1 eV in fixed transmission mode, and Phoibos 150 XPS spectrometer (SPECS) and Dealy Line Detector (Surface Concept). The spectra were fitted by CasaXPS software, using Shirley's background and Gaussian/Lorentzian line shape.

### 4.6 Electrolyte Recycling

To secure an adequate amount of electrochemically aged electrolyte for the subsequent recycling process, the full cells composed of HC anode and P2‐AFMNO layered oxide cathode, combining the pristine 0.8 m NaDFOB + 0.2 m NaFSI in GVL electrolyte, were assembled. Following the previous methodology, the X‐type Swagelok cells [31, 54] were used to electrochemically age the electrolyte in full cell configurations. The X‐cell design, accommodating 10 mm electrodes and a modified polyether ether ketone (PEEK) sleeve in place of a conventional glass fiber separator, allowed for an electrolyte reservoir of up to 1.5 mL in a single cell. To age the electrolyte, the full cells were cycled between 1.5 and 4.3 V. The cycling started with a formation cycle at 0.05C, followed by 4 cycles at 0.1C, and concluded with long‐term stability testing at 0.5C for 200 cycles. Upon completion of electrochemical aging in X‐cells, the electrolyte was then recovered inside an argon‐filled glovebox.

The electrolyte recycling process began by mixing 1 mL of electrolyte (aged electrolyte) with 1 mL of distilled water and 50 mL of a binary organic solvent mixture (cyclohexane/ethyl acetate, 1:1 v/v) in a separatory funnel. After thorough mixing and phase separation, the aqueous layer was collected. An additional 2 mL of distilled water was then added to the remaining organic phase, mixed, and allowed to separate for 30 min before recovering the aqueous layer again. This extraction procedure was repeated three times. The combined organic phases were concentrated by rotary evaporation (50°C, 145 mbar) to remove volatile solvents, forming a GVL‐rich fraction that was further purified overnight under vacuum using a Schlenk line to eliminate residual EtOAc, c‐Hex, and water. The recovered GVL was pre‐dried over 3 Å molecular sieves for three days prior to use. The residual water content was determined by Karl Fischer titration to be 4 ppm. The combined aqueous extracts, containing NaDFOB and NaFSI, were concentrated under reduced pressure using a Büchi Rotavapor R‐215. Details of the salts and organic solvents used for NMR analysis are provided in the Supporting Information.

## Conflicts of Interest

The authors declare no conflict of interest.

## Supporting information




**Supporting File**: smll72937‐sup‐0001‐SuppMat.docx.

## Data Availability

The data that support the findings of this study are available from the corresponding author upon reasonable request.
