# Supplementary material for: Development of Low Fluorinated, Sustainable, and Recyclable Electrolytes Based on γ‐Valerolactone for High‐Performance Sodium‐Ion Batteries
Source: Small. 2026 Feb 22;22(23):e00061. doi: 10.1002/smll.202600061 (PMC13100556; doi:10.1002/smll.202600061)
Supplement: Supplementary file 1 — Supporting File: smll72937‐sup‐0001‐SuppMat.docx. [file SMLL-22-e00061-s001.docx]

**Development of low fluorinated, sustainable and recyclable electrolyte based on γ-Valerolactone for high performance Sodium-Ion Batteries**

*Yiyue Lu^1†^, Muhammad Nouman Aslam^1†^, Juan Luis Gómez Urbano^1^, Shuting Zhang^2,3^, Maider Zarrabeitia ^2,3^, Timo Werner ^4^, Peter Axmann^4^, Christian Leibing^1^ and Andrea Balducci^1*^*

*^1^ Institute of Technical and Environmental Chemistry, Friedrich Schiller University Jena and Center for Energy and Environmental Chemistry (CEEC) Jena, Philosophenweg 7a, 07743 Jena, Germany*

*^2^ Helmholtz Institute Ulm (HIU), Helmholtzstrasse 11, 89081 Ulm, Germany*

*^3^ Karlsruhe Institute of Technology (KIT), P.O. Box 3640, 76021 Karlsruhe, Germany*

*^4^ ZSW Center for Solar Energy and Hydrogen Research Baden-Württemberg, Meitner-Strasse 24, 89081, Ulm, Germany*

**Corresponding author: andrea.balducci@uni-jena.de*

*^†^Yiyue Lu* (Author 1) and *Muhammad Nouman Aslam* (Author 2) contributed equally to this work.





***Figure S1*** *(a) Ionic conductivity between -30 to +80 °C; (b) Thermogravimetric analysis (TGA) tests performed to pristine* *sodium bis(fluorosulfonyl)imide (NaFSI) and sodium difluoro(oxalato)borate (NaDFOB) salt; (c) The isothermal TGA measurements of the three electrolytes at 60 °C.*





***Figure S2*** *Current evolution response from the anodic dissolution tests (a) 1 M NaFSI in γ-Valerolactone (GVL); (b) Comparison of the electrolytes containing NaDFOB.*

*
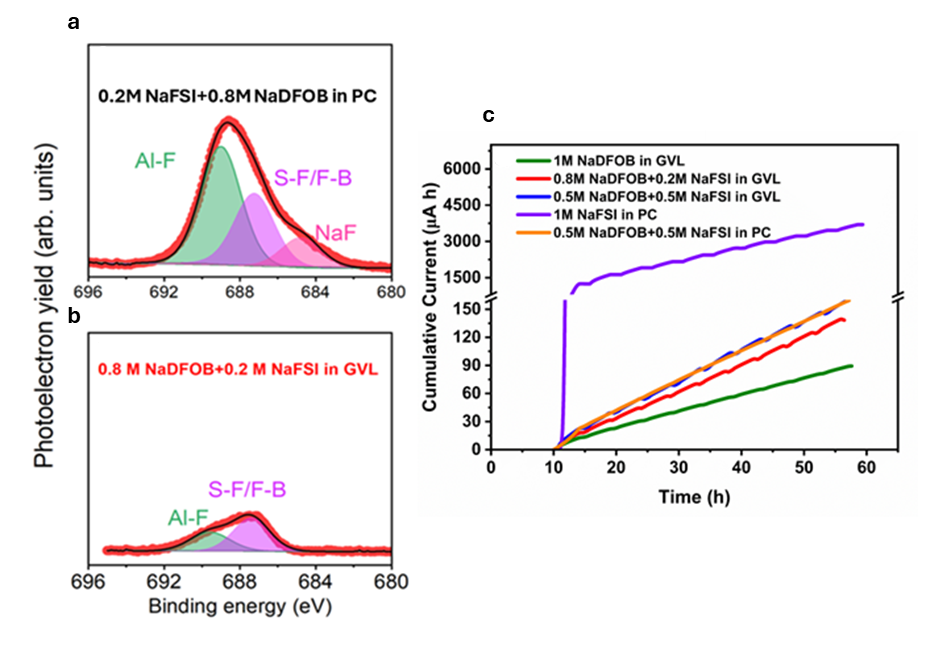
*

***Figure S3*** *XPS spectra of F 1s photoelectron region of cycled Al current collectors from the anodic dissolution tests in different electrolytes: (a) 0.8 M NaDFOB + 0.2 M NaFSI in propylene carbonate (PC) and (b) 0.8 M NaDFOB + 0.2 M NaFSI in GVL; and (c) Comparison of anodic dissolution test for the electrolytes based on Propylene carbonate (PC) and GVL.*

*

*

***Figure S4*** *Galvanostatic charge profiles registered for the first cycle of 1 M NaFSI in GVL at 0.1C.*

Although the 1 M NaFSI in GVL exhibits high ionic conductivity, it does not enable stable operation in P2-Na_2/3_Al_1/9_Fe_1/9_Mn_2/3_Ni_1/9_O_2_ (P2-AFMNO) half-cells. Notably, no reliable electrochemical response was obtained even when the upper cut-off voltage was limited to 3.8 V vs. Na^+^/Na, indicating severe incompatibility of the 1 M NaFSI in GVL with the P2-AFMNO half-cells under the tested conditions.





***Figure S5*** *Galvanostatic charge-discharge profiles registered for the first cycle of GVL-based electrolytes at (a) 0.1C and (b) 1C.*

At 0.1C, all three electrolytes show broad sloping plateaus during charge and discharge, occurring over approximately 3.3–4.1 V and 3.8–3.3 V vs. Na⁺/Na, respectively, indicating reversible sodium-ion storage processes. 0.5 M NaFSI + 0.5 M NaDFOB showed minimal voltage hysteresis, indicating reduced polarization and improved interfacial dynamics. The electrolytes containing NaFSI exhibited a slightly higher capacity than 1 M NaDFOB in GVL, which indicates that the increased amount of NaFSI in electrolyte composition not only improved transport properties such as higher ionic conductivity and low viscosity but also facilitated interfacial stability. The initial coulombic efficiency (ICE) can reach over 96% in the electrolytes containing NaFSI and stabilize at around 99% from the second cycle, which proves that the cell has higher reversibility and interface stability with good cycle performance.

At 1C, the differences between the various electrolytes become even more pronounced. The 1 M NaDFOB electrolyte exhibits significant polarization, which is evident from the lowest achieved capacity. In contrast, the electrolyte formulations consisting of dual-salt delivered high capacity, demonstrating significantly better performance in terms of rate capability. It should be noted that the 0.5 M NaDFOB + 0.5 M NaFSI in GVL formulation achieved the best capacity retention and minimal polarization among three electrolytes, confirming that the NaFSI improves ion transport and promotes the formation of a more ionically conductive and stable cathode-electrolyte interphase (CEI) layer.





***Figure S6*** *Galvanostatic charge-discharge voltage profiles of noted electrolytes in (a) 1^st^ cycle and (b) 100^th^ cycle at 0.5C.*

The voltage curves of the first and 100^th^ cycles clearly demonstrate the long-term stability of the three electrolyte systems. The electrolyte containing NaFSI shows a stable plateau even after 100 cycles, demonstrating excellent reversibility of the cell and stable interfacial behavior. On the other hand, the 1 M NaDFOB in GVL shows significant distortion of the plateaus and a markedly increased voltage hysteresis, indicating higher polarization and interfacial resistance.

The dual-salts electrolytes showed a much lower capacity drop between the first and the hundredth cycle compared to the single salt electrolytes, whereas the 1 M NaDFOB in GVL suffered severe capacity decreased. The addition of NaFSI can form a stable and thin CEI layer with better ion transport during long-term cycling.


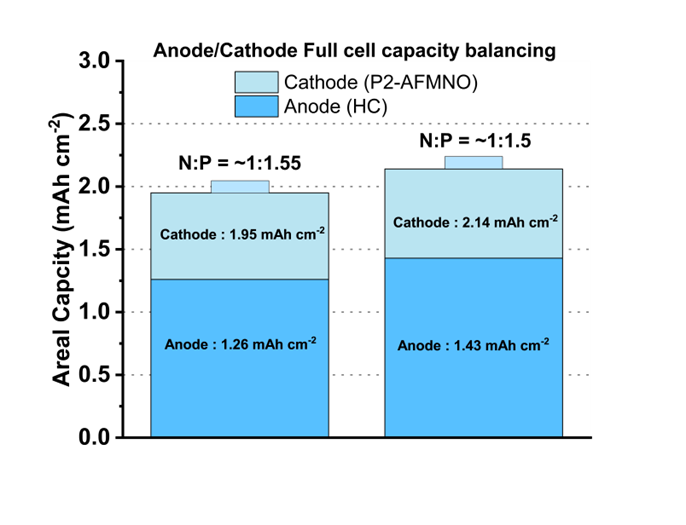


***Figure S7*** *Infographic representation of the areal capacities of the individual electrodes and resulting capacity balancing (N/P ratios) of the investigated full cells. The calculations for the areal capacities and (N/P ratio) balancing are based on specific capacities of 200 mAh g^-1^ for the hard carbon (HC) and 100 mAh g^-1^ for the layered oxide* *P2-Na_2/3_Al_1/9_Fe_1/9_Mn_2/3_Ni_1/9_O_2_ (P2-AFMNO) cathode.*


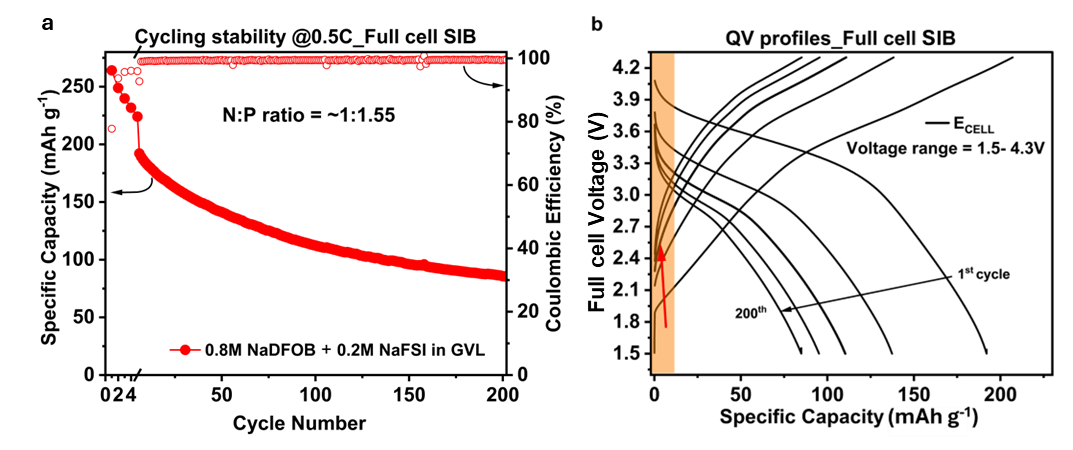


***Figure S8*** *Electrochemical performance of HC || P2-AFMNO cathode full cell (N:P=~1:1.55), (a) long cycling stability test conducted at 0.5C (after 1st formation cycle at 0.05C and 4 cycles at 0.1C), (b) Evolution of cell voltage vs. capacity profiles during the long cycle stability test at 0.5C over 200 cycles (1st, 50^th^,100^th^,150^th^,200^th^), using 0.8 M NaDFOB + 0.2 M NaFSI in GVL electrolyte.*


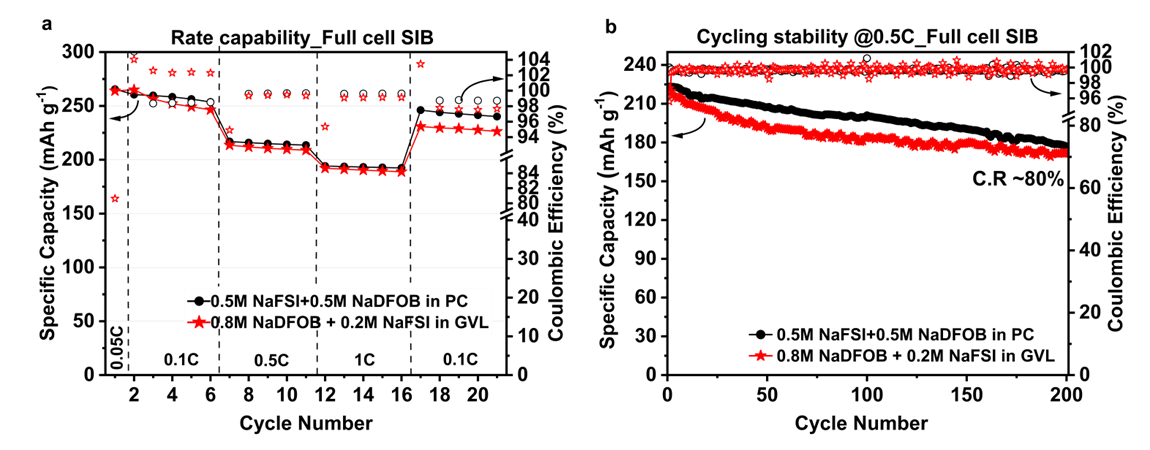


***Figure S9*** *A comparison of investigations on electrochemical performance of HC || P2-AFMNO cathode full cell (N:P=~1:1.5), (a) rate performance evaluation (1st formation cycle at 0.05C followed by 5 cycles of charging/discharging per each C-rate), (b) long cycling stability test conducted at 0.5C (after 1st formation cycle at 0.05C and 4 cycles at 0.1C ), using 0.8 M NaDFOB + 0.2 M NaFSI in GVL (red) and 0.5 M NaDFOB + 0.5 M NaFSI in PC (black) electrolyte, C.R= Capacity retention measured for 1^st^ cycle vs. 200^th^ cycle at 0.5C*


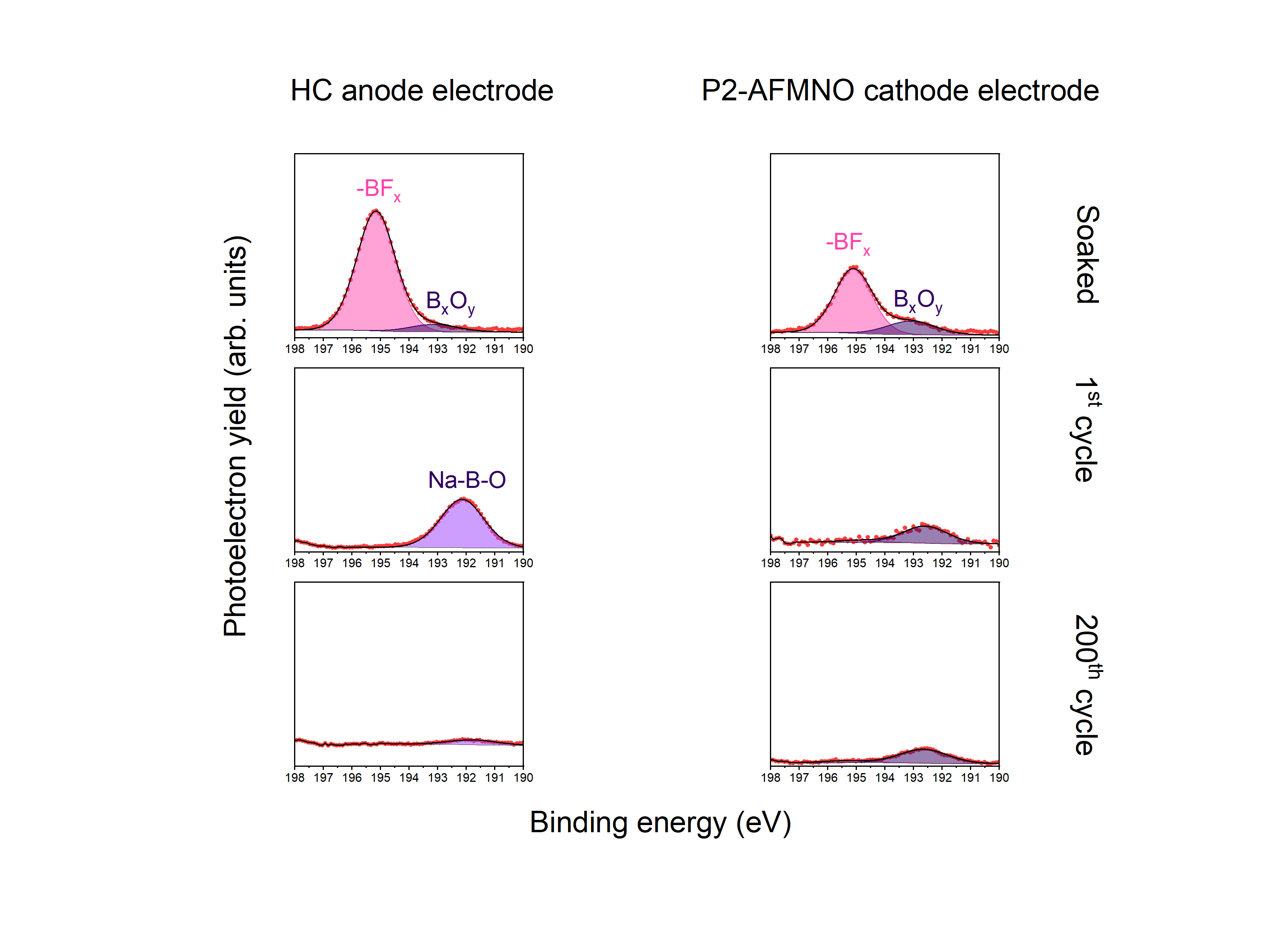


***Figure S10*** *B 1s photoelectron region of HC and P2-AFMNO electrodes: (top) soaked and cycled after the (middle) 1^st^ and (bottom) 200^th^ cycles.*


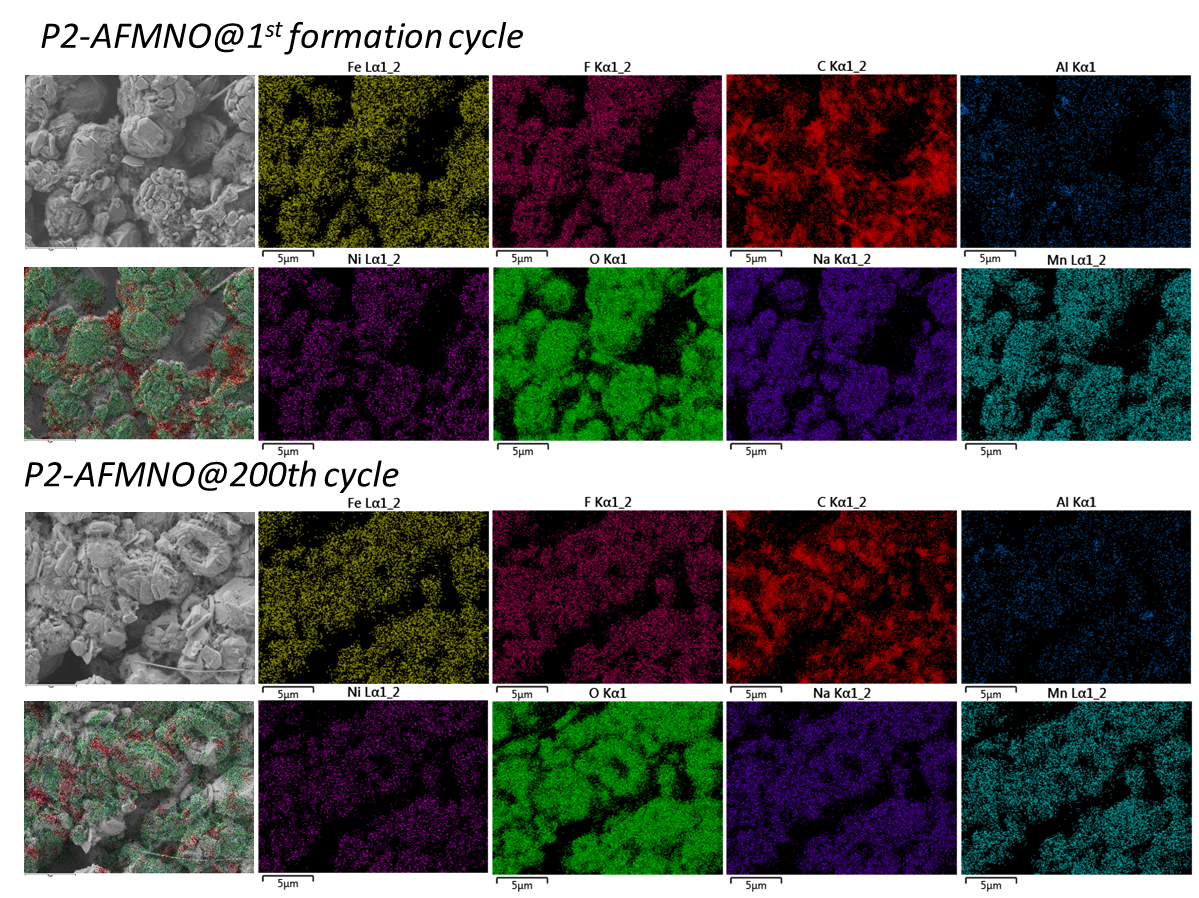


***Figure S11*** *Elemental distribution of the cycled P2-AFMNO electrodes in the full cell HC || P2-AFMNO.*

**
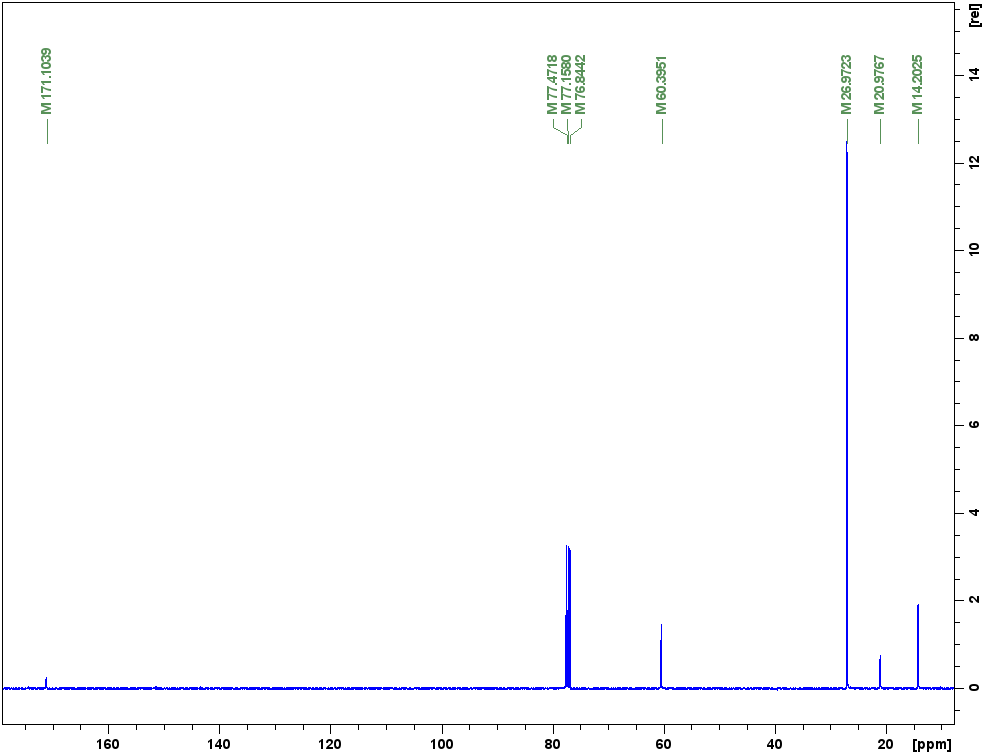
**

**a**

^13^C Votatile phase

(EtOAC-c-Hex phase)

**b**


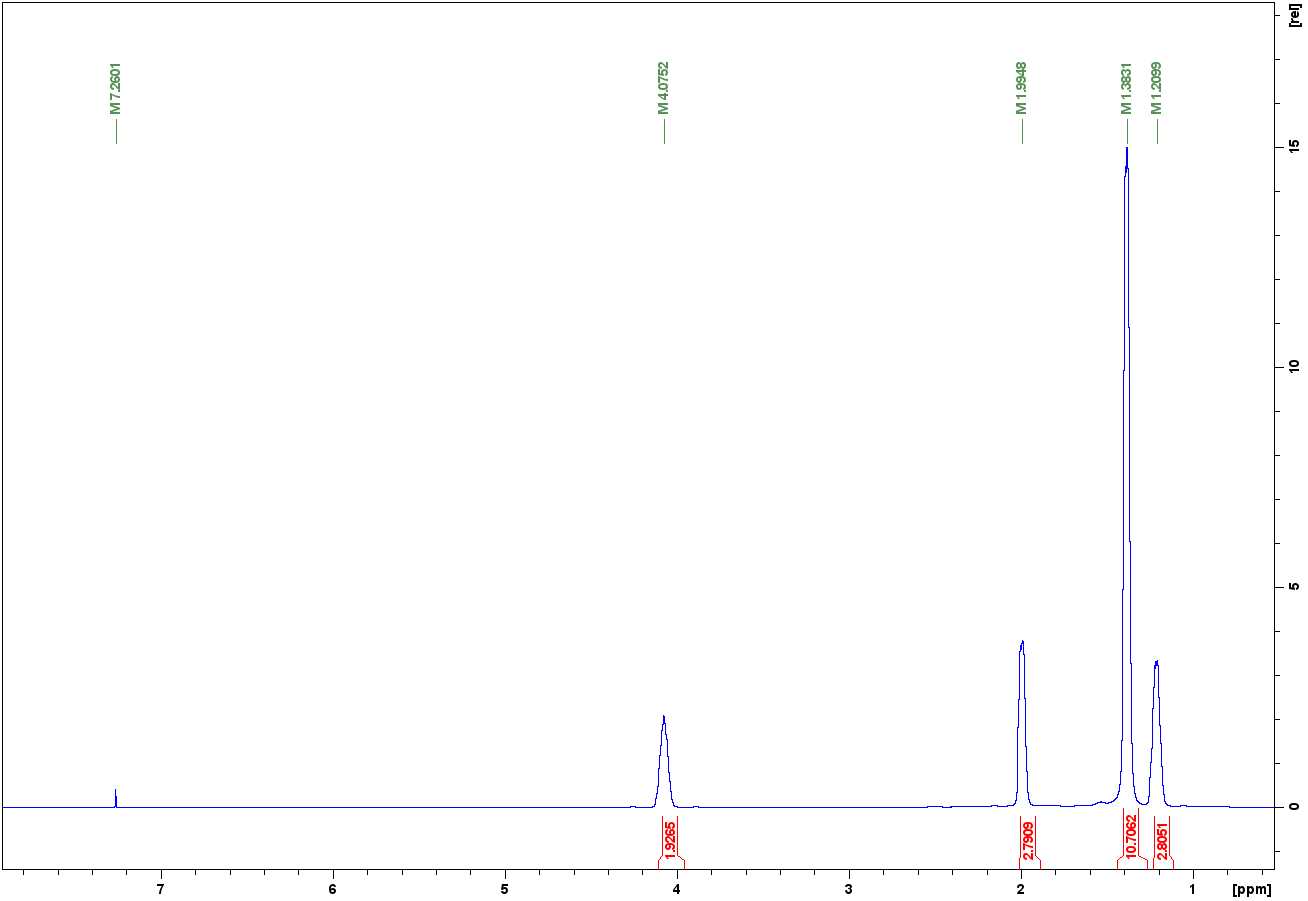


^1^H Votatile phase

(EtOAC-c-Hex phase)


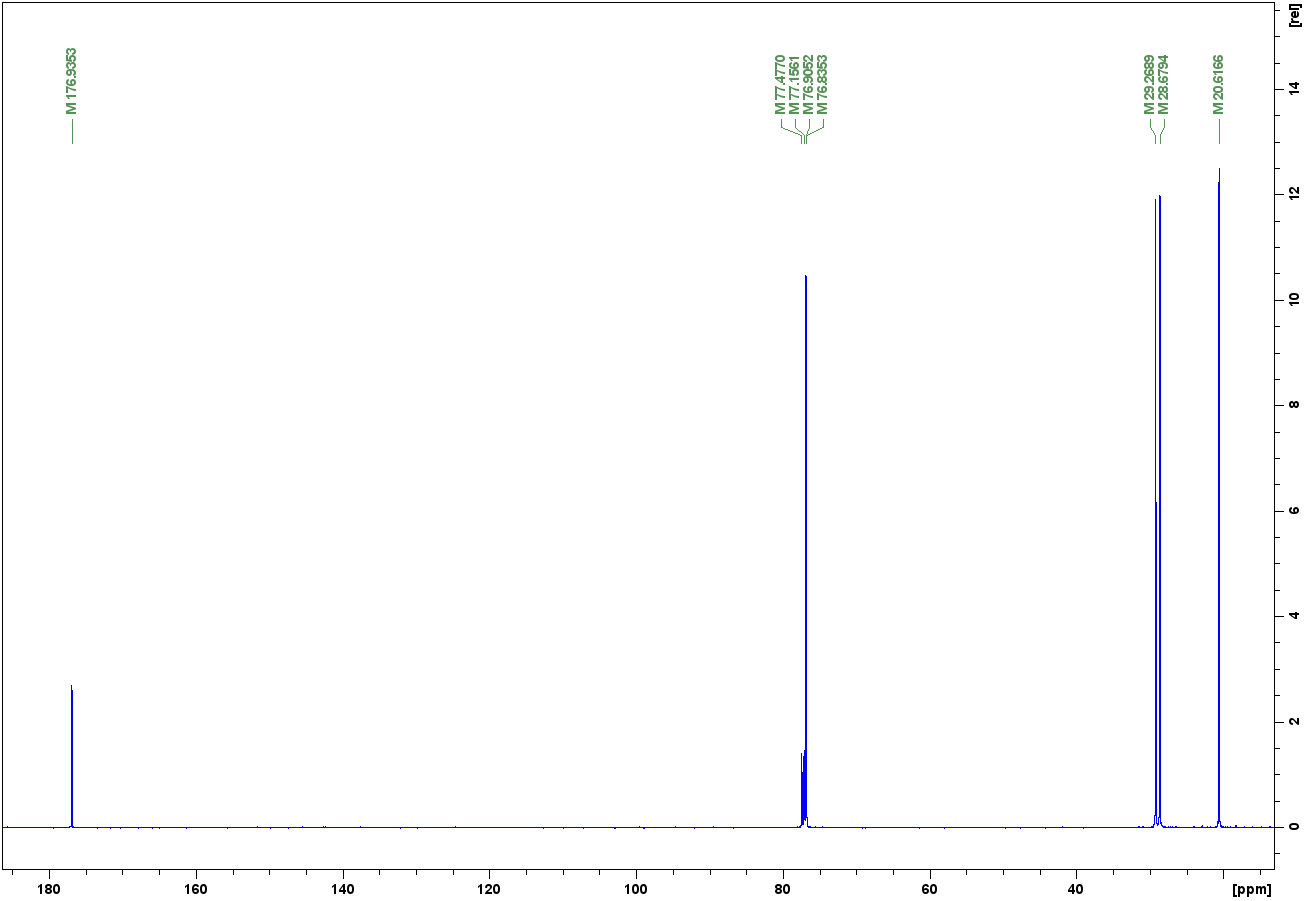


^13^C Distillation tail

(GVL phase)

**c**

c

**d**


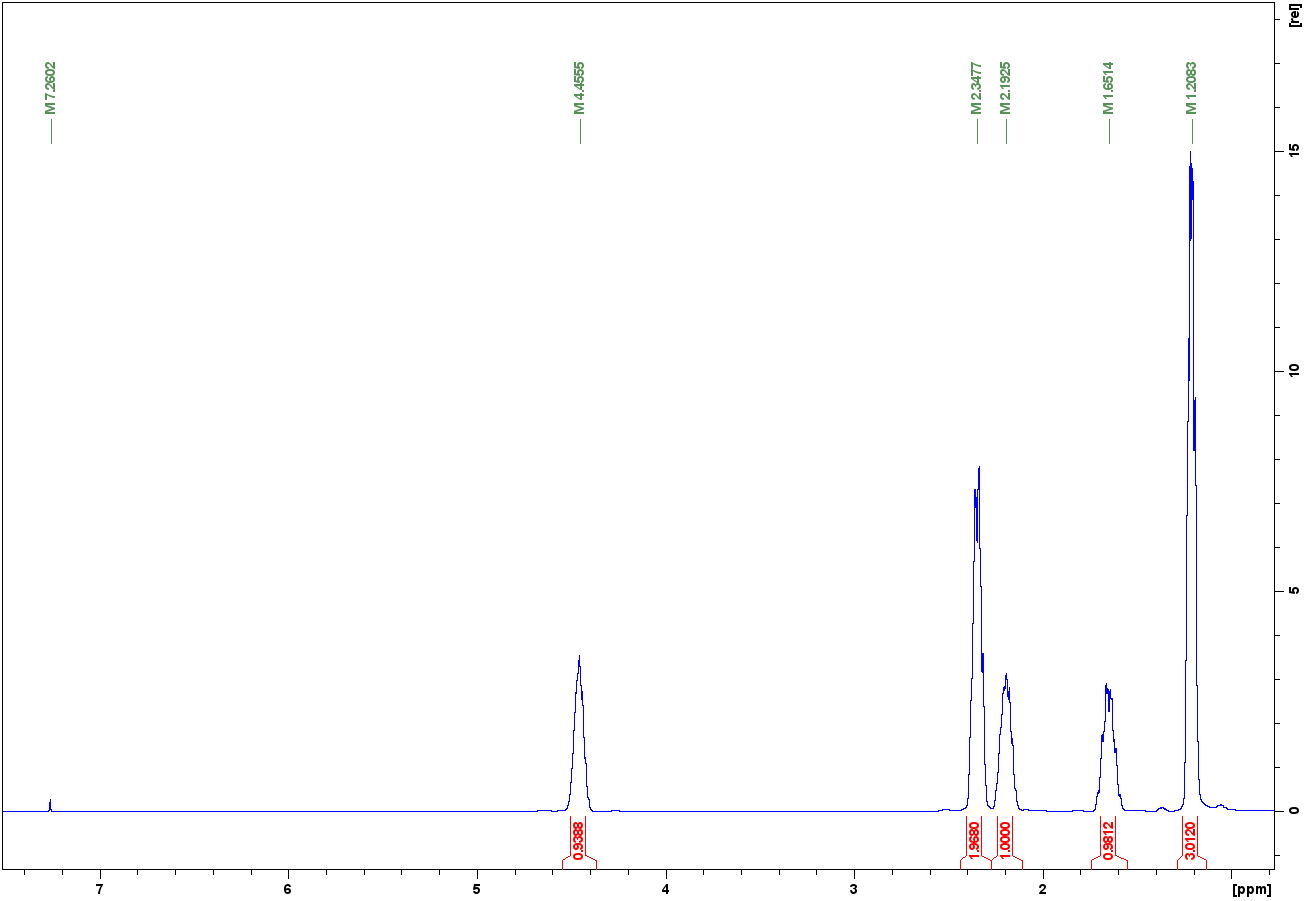


^1^H Distillation tail

(GVL phase)

***Figure S12*** *(a) ^13^C NMR portions of distilled volatile phase; (b) ^1^H NMR portions of distilled volatile phase. Found molar ratio: c-Hex 55 %mol; EtOAc 45 %mol vs. theoretical molar ratio for 1:1 in volume solution: c-Hex 52%mol; EtOAc 48%mol. (c) ^13^C NMR of recovered GVL phase (distillation tails) in CDCl_3_; (d) ^1^H NMR of recovered GVL phase (distillation tails) in CDCl_3_.*

NMR spectra were recorded on Bruker Avance II (400.13 MHz, 1H; 100.62 MHz, 13C) with a 5mm PA BBF-H-D-05 Z probehead. Measurements were performed in deuterated CDCl₃. For ¹H measurements, the chemical shifts were referenced to added CDCl₃ (δ = 7.26 ppm), For ^13^C measurements, the chemical shifts were referenced to added CDCl₃ (δ = 77.16 ppm, triplet peak). The spectra were analyzed using Bruker TopSpin software. The volume ratio of the distillate was calculated based on the NMR data and compared with the corresponding theoretical value. Slight deviations in chemical shifts and the absence of resolvable coupling constants are attributed to signal broadening caused by the relatively high analyte concentration and the complex sample matrix of the recycled solvent.

(a)^13^C NMR of organic phase δppm (101 MHz): 14.2 (EtOAc) ,20.9 (EtOAc), 26.9 (c-Hex), 60.4 (EtOAc), 177.1 (EtOAc)

(b)^1^H NMR of organic phase δppm (400 MHz): 1.21 (s, 3H, EtOAc), 1.38 (br m, 12H, c-Hex), 1.99 (br s, 3H, EtOAc), 4.07 (br, s, 2H, EtOAc).

(c) ^13^C NMR of GVL δppm (101 MHz): 20.6, 28.7, 29.3, 78.8, 177.9

(d) ^1^H NMR of GVL δppm (400 MHz): 1.21 (br s, 3H), 1.65 (br m, 1H), 2.19 (br m, 1H), 2.35(br m, 2H), 4.45 (br s,1H).

KF (water content) of recovered GVL: 4ppm





***Figure S13*** *TGA analysis of the pristine and recovered GVL.*


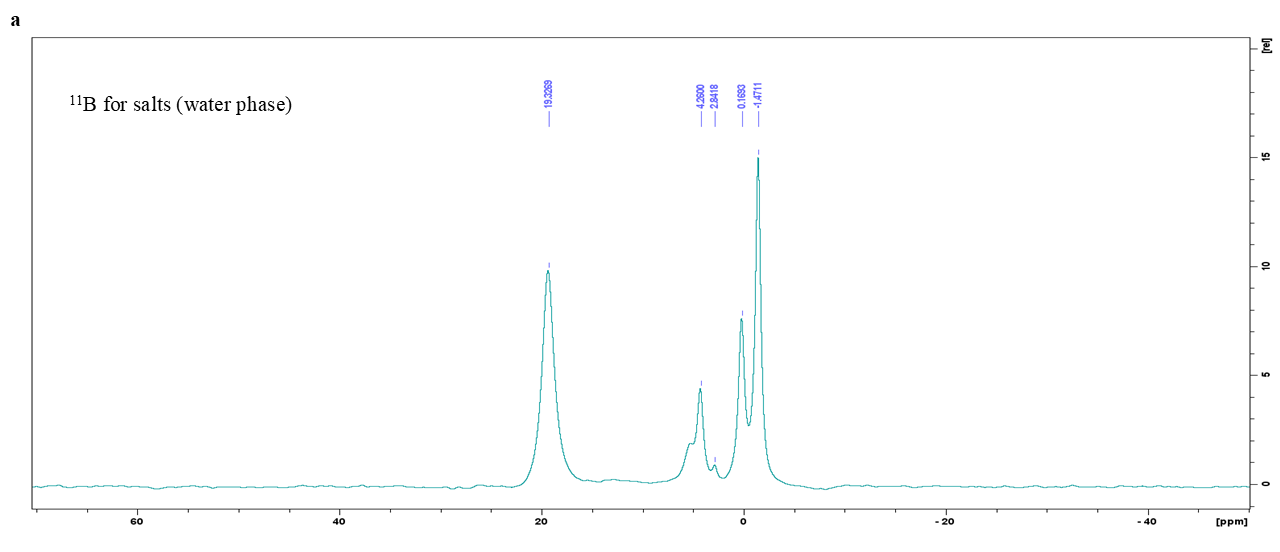


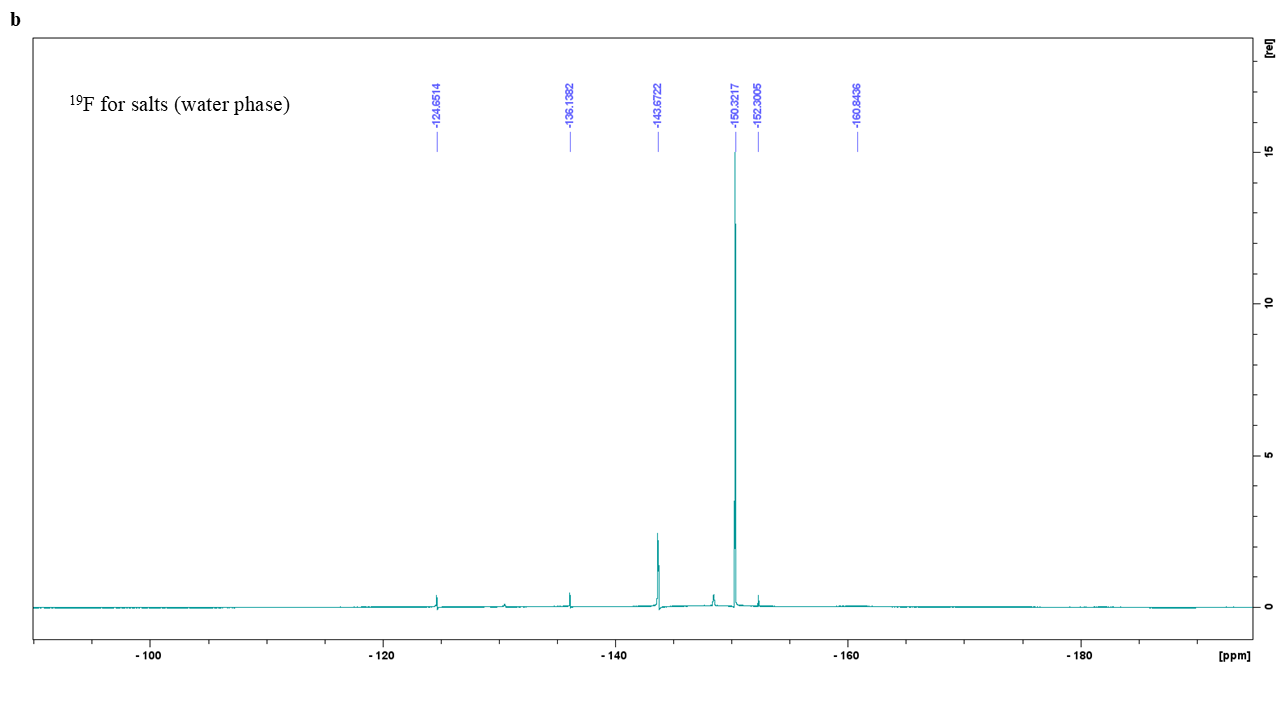


***Figure S14*** *(a) ^11^B NMR and (b)^19^F NMR portions of salts in water phase.*

NMR spectra were recorded on Bruker Avance NEO 500 CPP BBFO Prodigy probehead. Measurements were performed in deuterated DMSO. The spectra were analyzed using Bruker TopSpin software.

(a)^11^B NMR of Salts δppm (160 MHz): 19.3, 4.3, 2.9, 0.2, -1.5

(b)^19^F NMR of Salts δppm (470 MHz): -124.4, -136.1, -143.7, -150.3, -152.3, -160.4


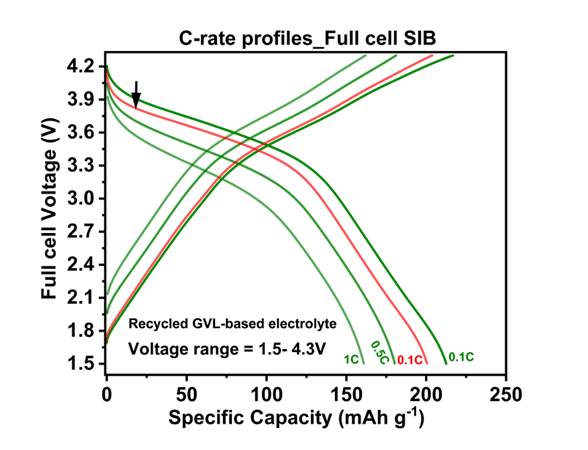


***Figure*** ***S15*** *C-rate profiles at different applied currents of HC || P2-AFMNO full cell (N:P=~1:1.5) using 0.8 M NaDFOB + 0.2 M NaFSI in “Recycled GVL” electrolyte.*

**Cathode material synthesis**

The layered double hydroxide (or hydrotalcite-type) precursor [Al_1/9_Fe_1/9_Mn_2/3_Ni_1/9_(OH)_2_]^+2/9^[(NO_3_^-^)_2/9_ ∙ z H_2_O]^-2/9^ was synthesized by continuously feeding water, a deaerated solution of NaOH (Carl Roth), and a stoichiometric deaerated solution of Al(NO_3_)_3_ x 9 H_2_O, Fe(NO_3_)_3_ x 9 H_2_O, Mn(NO_3_)_2_ x 4 H_2_O and Ni(NO_3_)_2_ x 6 H_2_O (all Carl Roth) into the vigorously stirred tank reactor (CSTR, Volume 1 liter). Citric acid added to the metal solution served as chelating agent.^[1]^After the run-in period, the dispersion at the reactor outlet was collected and continuously washed and filtered to remove any residual salt solution. The dried precipitate was heated in a muffle furnace (Nabertherm) at 900 °C for 1 h in air to remove any crystal water from the hydrotalcite-type precursor transforming the precursor to an oxide. Subsequently, the precursor was dry mixed with a respective amount of Na_2_CO_3_ (Sigma Aldrich) and then heated in a box furnance (Carbolite Gero) at 950 °C for 10 h in synthetic air (20 vol.% O_2_ in Ar). After natural cooling to 200 °C, the obtained powder was directly transferred into a Büchi glass oven, where it was kept at 200°C and dynamic vacuum (~ 2 x 10^-2^ Pa) overnight. The obtained cathode active material was then relocated without any further contact with ambient air into an Ar-filled glovebox (MBraun, O_2_ < 0.1 ppm, H_2_O < 0.1 ppm), where subsequent powder handling and electrode preparation was performed.

**Characterization of cathode active material**

Elemental analysis of the obtained cathode active material was performed using inductively coupled plasma optical emission spectroscopy (ICP-OES, Spectro Arcos SOP) with a diluted *aqua regia* solution. The obtained chemical composition represented as a chemical formula for layered sodium transition metal oxides (Na_x_MO_2_ with M = Al, Fe, Mn, Ni) is Na_0.659_Al_0.115_Fe_0.113_Mn_0.660_Ni_0.113_O_2_, which is in good accordance with the targeted stoichiometry of Na_2/3_Al_1/9_Fe_1/9_Mn_2/3_Ni_1/9_O_2_. The crystal structure of the cathode active material was characterized using X-ray powder diffraction in Bragg-Brentano geometry on a Bruker D8Advance with a Cu X-ray source and a LynxEye XE-T detector. The obtained diffraction pattern is presented in Figure S16. The obtained diffraction pattern is similar to PDF 00-054-0839 and is well indexed with the hexagonal space group *P*6_3_/*mmc* (SG 194), indicating the phase pure P2-type^[2]^ crystalline structure. The morphology of the cathode active material was depicted using scanning electron microscopy on a Zeiss Leo 1530VP equipped with an Everhart-Thornley SE detector at 5 kV acceleration voltage as presented in Figure S17. The cathode active material consists of dense, round secondary particles in the range of 2 µm to 15 µm.


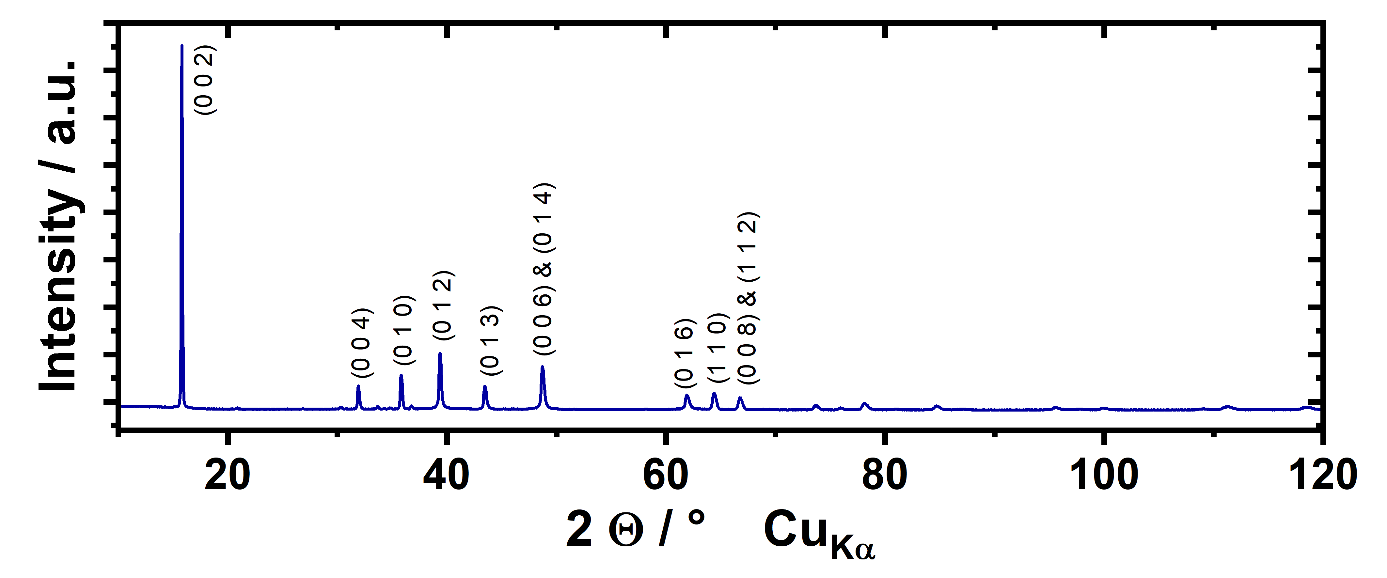
***Figure S16*** *Powder X-ray diffraction pattern of the cathode active material.*


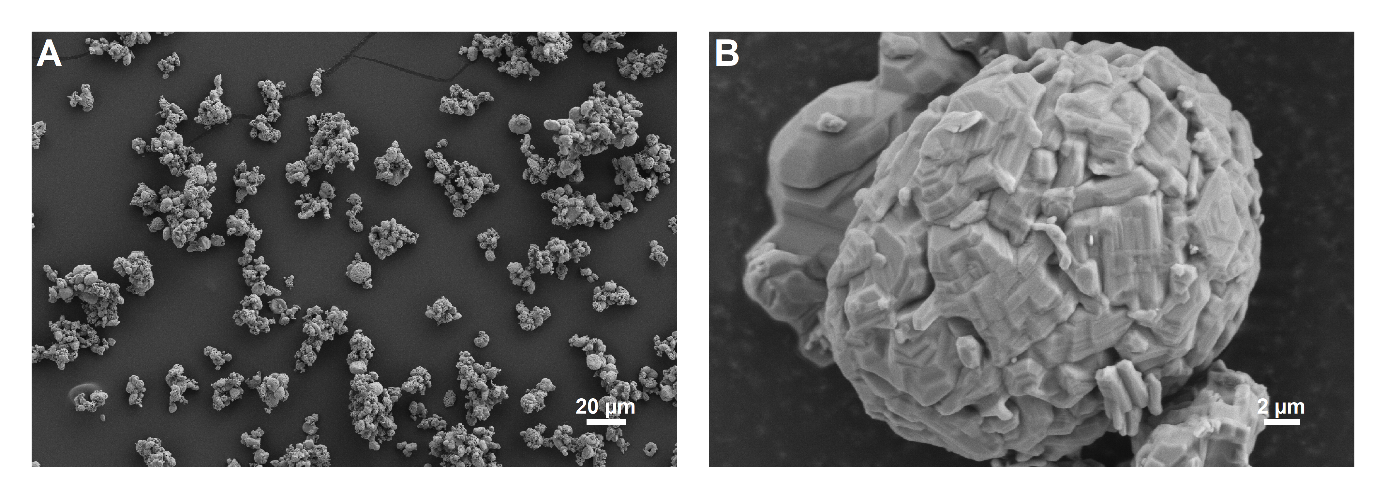
***Figure S17*** *SEM images of the as-prepared cathode active material.*


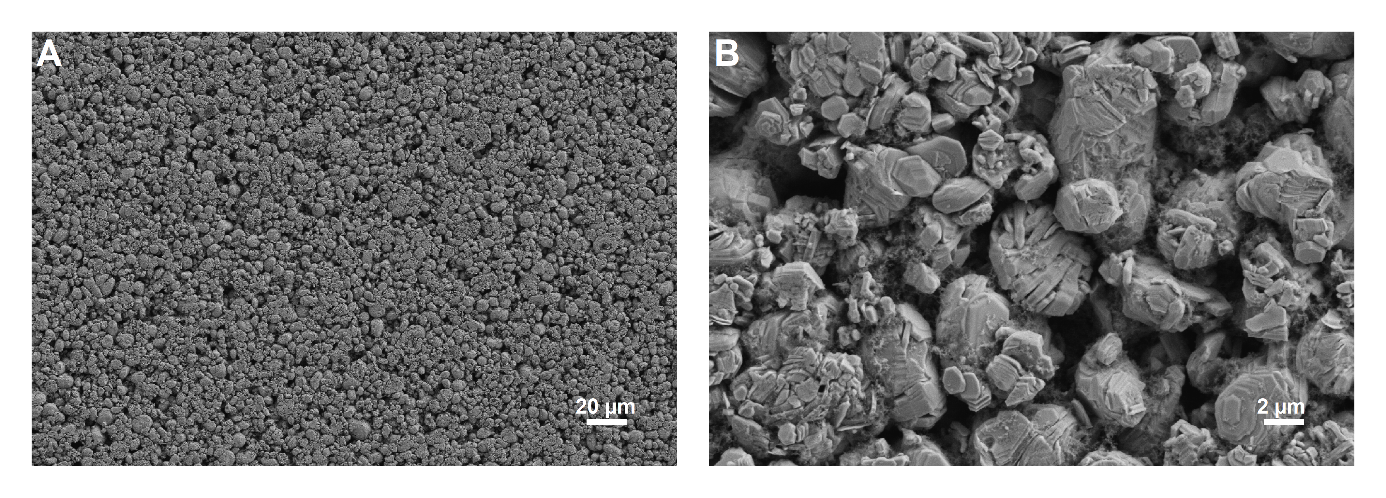


***Figure S18*** *SEM images of the cathode electrode as a top-view.*

**
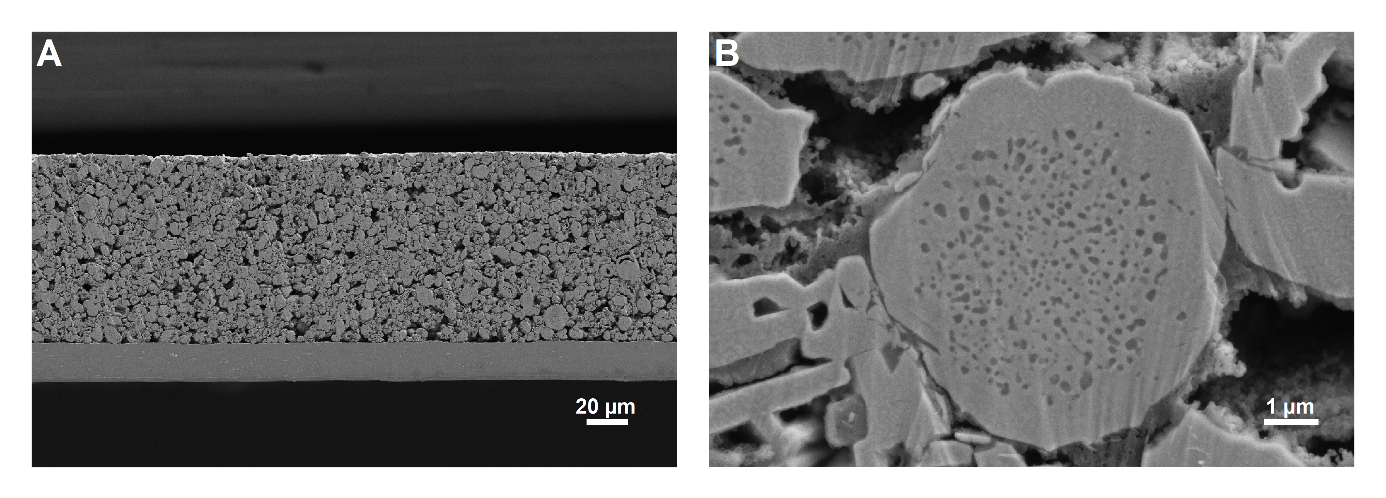
*Figure S19*** *SEM images of the cathode electrode as cross-section.*

References

1 J. Lamb and A. Manthiram, "Synthesis Control of Layered Oxide Cathodes for Sodium-Ion Batteries: A Necessary Step Toward Practicality," *Chem. Mater.* **2020**, *32* (19), 8431–8441, https://doi.org/10.1021/acs.chemmater.0c02435.

2 C. Delmas, C. Fouassier,and P. Hagenmuller, "Structural classification and properties of the layered oxides," *Physica B+C* **1980**, *99* (1-4), 81–85, https://doi.org/10.1016/0378-4363(80)90214-4.
